# A C2HC zinc finger is essential for the RING-E2 interaction of the ubiquitin ligase RNF125

**DOI:** 10.1038/srep29232

**Published:** 2016-07-14

**Authors:** Marie-José Bijlmakers, João M. C. Teixeira, Roeland Boer, Maxim Mayzel, Pilar Puig-Sàrries, Göran Karlsson, Miquel Coll, Miquel Pons, Bernat Crosas

**Affiliations:** 1 Institut de Biologia Molecular de Barcelona, CSIC, Parc Cientific de Barcelona, 08028 Barcelona, Spain; 2 BioNMR Laboratory, Inorganic and Organic Chemistry Department, Organic Chemistry Section, University of Barcelona, Parc Cientific de Barcelona, 08028 Barcelona, Spain; 3 XALOC beamline, ALBA synchrotron (CELLS), 08290 Cerdanyola del Valles, Spain; 4 Swedish NMR Centre, University of Gothenburg, SE-40530, Gothenburg, Sweden; 5 Institute for Research in Biomedicine (IRB Barcelona), Parc Cientific de Barcelona, 08028 Barcelona, Spain

## Abstract

The activity of RING ubiquitin ligases (E3s) depends on an interaction between the RING domain and ubiquitin conjugating enzymes (E2), but posttranslational events or additional structural elements, yet largely undefined, are frequently required to enhance or regulate activity. Here, we show for the ubiquitin ligase RNF125 that, in addition to the RING domain, a C2HC Zn finger (ZnF) is crucial for activity, and a short linker sequence (Li2^120-128^) enhances activity. The contribution of these regions was first shown with truncated proteins, and the essential role of the ZnF was confirmed with mutations at the Zn chelating Cys residues. Using NMR, we established that the C2HC ZnF/Li2^120-128^ region is crucial for binding of the RING domain to the E2 UbcH5a. The partial X-ray structure of RNF125 revealed the presence of extensive intramolecular interactions between the RING and C2HC ZnF. A mutation at one of the contact residues in the C2HC ZnF, a highly conserved M112, resulted in the loss of ubiquitin ligase activity. Thus, we identified the structural basis for an essential role of the C2HC ZnF and conclude that this domain stabilizes the RING domain, and is therefore required for binding of RNF125 to an E2.

The modification of proteins by ubiquitination is a versatile mechanism that regulates almost every cellular process either through the destruction of target proteins or the spatial or temporal alteration of their functions^1^. The specificity of this posttranslational modification is determined by the ubiquitin ligases or E3s that recruit the substrates to which ubiquitin becomes covalently attached^2^. Consistent with this role, the E3s comprise a large diverse group of proteins that share the ability to interact with an E2, or ubiquitin conjugating enzyme, via a domain that is most frequently a RING, U-box or HECT domain^3^. Mechanistically, the E3s fall into two categories, those that covalently bind ubiquitin before transferring it to a substrate, as is the case for the HECT and the RING RBR E3s, and those that do not form a covalent ubiquitin-intermediate, as is the case for the U-box proteins and the majority of the RING E3s. Of these, the RING domain proteins are the largest group with more than 500 highly diverse members encoded in mammalian genomes^4^.

Major insights into the mechanism by which RING E3s stimulate the transfer of ubiquitin from an E2 to a substrate without covalently binding ubiquitin themselves, have been obtained from structural studies of RING (and U-box) domains associated with ubiquitin-loaded E2s^5^,^6^,^7^. This demonstrated that RING domains exert allosteric effects on E2-Ub conjugates, stabilizing a closed conformation whereby ubiquitin is positioned on the E2 in a manner that is optimal for nucleophilic attack at the thioester bond by a lysine of the substrate^8^,^9^,^10^. Nevertheless, it is also clear that in many cases the presence of a RING domain is not sufficient and additional events are needed, or significantly enhance the function of ubiquitin ligases^11^. These regulatory mechanisms are still being defined, but various principles can be distinguished such as a requirement for homodimerization (e.g. RNF4, BIRC7, XIAP, and IDOL)^5^,^12^,^13^,^14^, heterodimerization (e.g. in Mdm2-MdmX, BRCA1-BARD1, RING1B-Bmi1)^15^,^16^,^17^,^18^, or the binding of small molecules (sphingosine-1-phosphate binding to TRAF2^19^ or PAR binding to RNF146)^20^. Additionally, a role has been described for auxiliary elements of E3s in enhancing ubiquitin release from the E2, either through binding to the E2 (e.g. for gp78^21^,^22^ and AO7)^23^ or to the donor ubiquitin on the E2 (e.g in Cbl)^24^.

To obtain new insights into regulatory structures of ubiquitin ligases, we have studied the RING protein RNF125 (previously called TRAC-1). RNF125 was first identified as a regulator of T cell activation^25^, but has also been shown to negatively regulate the cytoplasmic viral RNA sensors RIG-I and MDA5^26^, to interfere with HIV transcription^27^ and promote p53 degradation^28^. Furthermore, variations in *RNF125* gene expression levels have been linked to progression of HIV infection^29^, and resistance of melanoma cells to B-raf inhibitors^30^. Recently, mutations in RNF125 have been associated with a new overgrowth syndrome, characterized by macrocephaly as well as inflammatory disease^31^. These diverse roles of RNF125 remain to be further understood.

RNF125 is a small protein (25 kD) that contains, in addition to the RING domain, three zinc fingers (ZnFs) and a ubiquitin interacting motif (UIM) (Fig. 1A). So far, only the interactions via the RING and UIM with E2s and ubiquitin, respectively, have been demonstrated^32^. The *in vitro* ubiquitination activity of RNF125 is readily detectable and in cells, this activity correlates with low steady state levels and a short half-life^32^. RNF125 belongs to a protein family with three related members, RNF114, RNF138 and RNF166^32^ that, despite similarities in size, amino acid sequence and domain organization, differ considerably in activity. This is especially the case for RNF114, a psoriasis susceptibility gene product^33^,^34^, which has very low activity compared to RNF125^33^. This difference in activity between RNF125 and RNF114 was the starting point of our study and led, through the generation of chimeras and stop mutants, to the identification of an essential role of the C2HC ZnF in the activity of RNF125, and a contributing, non-essential role for a linker region. NMR experiments demonstrated that the establishment of RING interactions with the E2 depends on the presence of these auxiliary regions. X-ray crystallography showed interactions between the RING and C2HC ZnF, whilst mutations at one of these contact residues, M112, resulted in an inhibition of ubiquitin ligase activity, elucidating an important structural role of the C2HC ZnF. We conclude that the RING and C2HC ZnF form a functional unit that is required for binding to the E2.

## Results

### The RING domain of RNF125 is not sufficient for activity

**Figure 1:**
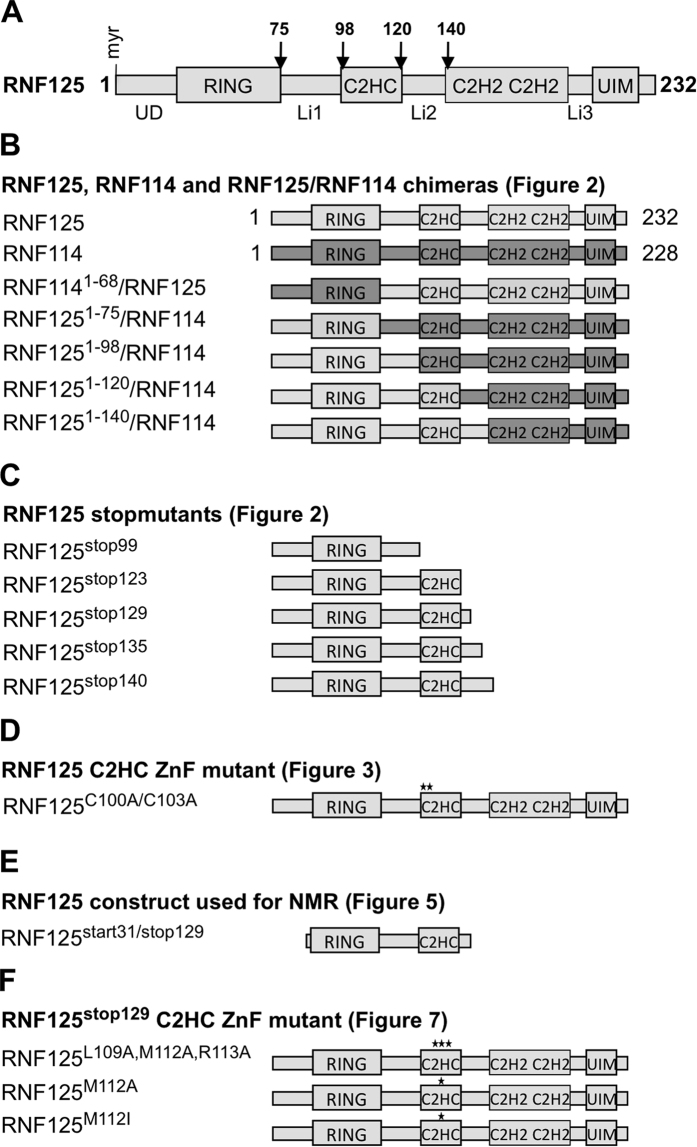
RNF125, RNF114 and the mutants used in this study. (**A**) Schematic representation of the RNF125 domain organization. Myr: myristoylation site; UD: unique domain, which is different for the four family members RNF114, RNF125, RNF138 and RNF166; Li1, Li2 and Li3 are linker regions; C2HC and C2H2 are Zn fingers. (**B**) Diagrams of RNF125, RNF114 and the chimeras of RNF125 and RNF114 used in Fig. 2. RNF125 regions are in light grey, RNF114 regions in dark grey. RNF114^1-68^/RNF125 contains amino acids 1-68 of RNF114 and the remainder, starting from the equivalent position of RNF114 residue 69, of RNF125; Similarly, RNF125^1-75^/RNF114 contains amino acids 1-75 of RNF125 and the remainder of RNF114, etcetera. (**C**) Diagrams of the RNF125 stop mutants used in Fig. 2. (**D**) The C2HC ZnF mutant used in Fig. 3. The stars indicate the Ala substitutions at Cys100 and Cys103. (**E**) The RNF125^start31/stop129^ protein used in the NMR experiments in Fig. 5. The protein starts at Pro31 and ends at Pro128. (**F**) The RNF125 mutants used in Fig. 7. The stars indicate mutations introduced at residues L109, M112 and R113 that, based on the crystal structure, make contacts with the RING domain.

**Figure 2:**
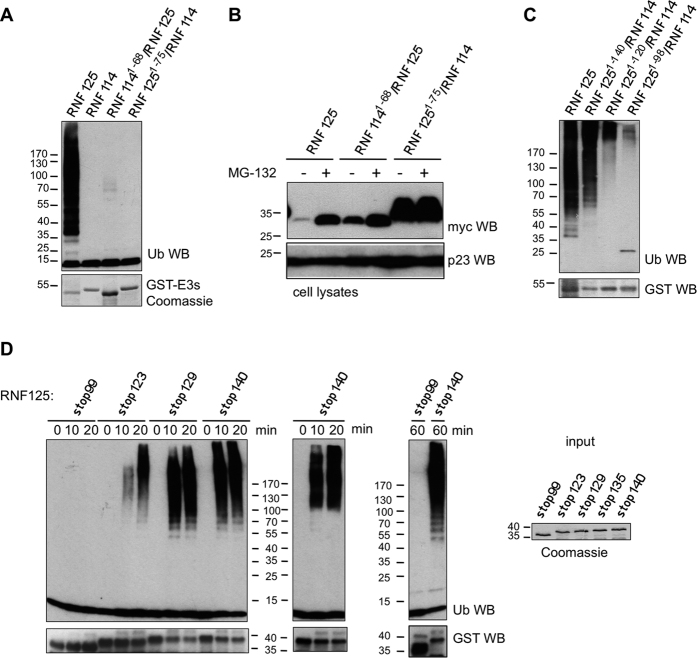
Ubiquitin ligase activity of RNF125/RNF114 chimeras and RNF125 stop mutants. (**A**) *In vitro* ubiquitination reactions with GST fusion proteins of RNF125, RNF114 and the chimeras, for 60 min at 37 °C, were analyzed by Western blotting (WB) with anti-ubiquitin antibody (Ub). Coomassie staining shows the input levels of E3s. (**B**) HEK293T cells transfected with cDNAs for myc-tagged RNF125 or the RNF125/RNF114 chimeras, were either left untreated (-) or treated with MG-132 (20 μM) for 2 h. Cell lysates were analyzed by Western blotting for RNF125 levels with anti-myc. Blotting for the chaperone protein p23 was used as a loading control. (**C**) *In vitro* ubiquitination reactions with RNF125 and the indicated chimeras, performed as in panel A. Ubiquitination was detected with anti-ubiquitin (Ub) and input levels of the chimeras are shown by anti-GST Western blotting. The band around 25 kD in the RNF125^1-98^/RNF114 lane (Ub WB) most likely represents a multimer of ubiquitin. (**D**) *In vitro* ubiquitination reactions with GST fusion proteins of RNF125 stop mutants were stopped at 0, 10 and 20 min (two panels on left). The reactions were analyzed with anti-ubiquitin antibody (Ub) to detect ubiquitination, and subsequently with anti-GST (GST) to detect the fusion proteins. Reactions of RNF125^stop99^ and RNF125^stop140^ were also run for 60 min. To show relative input levels of E3s, the GST fusion proteins were also loaded separately and stained with Coomassie (panel on the right).

We previously compared the *in vitro* ubiquitination activity of RNF125 to that of its relative RNF114 using proteins immunoprecipitated from transfected HEK293T cells^33^. These reactions, performed in the presence of UbcH5a/b/c as E2 proteins, showed significantly higher activity for RNF125 than for RNF114. Here, we performed these reactions with GST-fusion proteins purified from *Escherichia coli* and found an even greater difference, with the activity of RNF114 being virtually undetectable (Fig. 2A). This indicated that intrinsic features of the proteins are responsible for this difference. To compare RNF125 and RNF114 proteins further, we made chimeras, exchanging the N-terminal unique domain and RING domain of RNF114 with the same part of RNF125, and vice versa (Fig. 1B, see Supplementary Fig. S1 for a sequence comparison of RNF125 and RNF114). The chimera with the RING domain of RNF114, RNF114^1-68^/RNF125, showed very little *in vitro* ubiquitin ligase activity consistent with the lower activity of RNF114 (Fig. 2A). Surprisingly, the chimera with the RING domain of RNF125, RNF125^1-75^/RNF114, was also inactive despite this RING domain being indispensable for RNF125 activity^32^,^35^. Results in HEK293T cells using myc-tagged forms of the chimeras were consistent with the *in vitro* ubiquitination results. Both RNF114^1-68^/RNF125 and RNF125^1-75^/RNF114 were expressed at higher levels than RNF125 and were affected less by proteasome inhibition, suggestive of lower auto-ubiquitination activity in cells (Fig. 2B). Thus, it can be concluded that whereas the RNF125 RING domain is essential, it is not sufficient for ubiquitin ligase activity.

### The C2HC ZnF is crucial and linker 2 residues contribute to RNF125 activity

To investigate a role for the domains located to the C-terminus of the RING domain, the activity of additional RNF125/RNF114 chimeras (Fig. 1B) was analyzed. This showed that a chimera with the first 98 amino acids of RNF125, RNF125^1-98^/RNF114, had very little *in vitro* activity (Fig. 2C), but this was increased by the presence of an additional 22 amino acids in RNF125^1-120^/RNF114, and further by an adjacent 20 residues in RNF125^1-140^/RNF114. Thus, the region between residues 99 and 140 that contains the C2HC ZnF and linker 2 (Li2) (Fig. 1A) seemed to play an important role in RNF125 activity.

To exclude the alternative explanation that the RNF114 domains were inhibitory, we made RNF125 stop mutants with truncations at residues 99, 123 or 140 (Fig. 1C) and tested these in time course *in vitro* ubiquitination assays (Fig. 2D). Consistent with the results of the RNF125/RNF114 chimeras this showed that ubiquitin ligase activity was undetectable for RNF125^stop99^, a construct that contains only the RING domain and adjoining linker sequences. Activity was also not detected when the reaction was allowed to proceed to 60 min (Fig. 2D). However, the inclusion of the C2HC ZnF domain in RNF125^stop123^ resulted in increased activity, which was further enhanced by the addition of Li2 in RNF125^stop140^ (Fig. 2D). Two further stop mutants were made to define more precisely the region between 123 and 140 that enhances activity. Of these, RNF125^stop129^ showed increased activity compared to RNF125^stop123^, whereas no further increase was observed with RNF125^stop135^ (Fig. 2D). Taken together, the results with the chimeras and stop mutants demonstrate that the C2HC ZnF of RNF125 is essential for ubiquitin ligase activity, and that residues 120–128 of linker 2 (Li2^120-128^) further enhance activity.

### Point mutations at cysteines in the C2HC ZnF inhibit RNF125 activity

**Figure 3:**
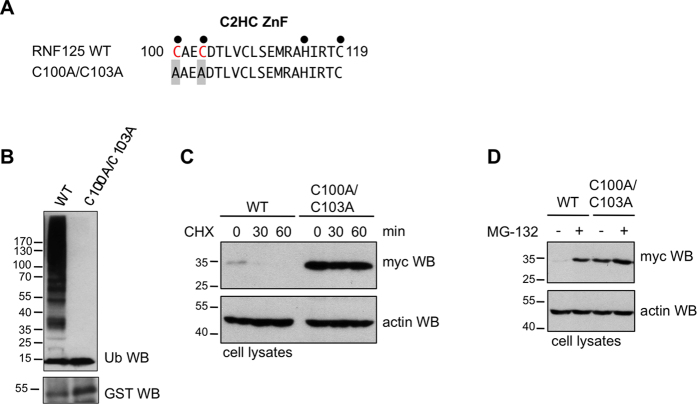
Effects of C2HC ZnF Cys mutations on RNF125 activity. (**A**) Amino acid sequence of the C2HC ZnF for RNF125 and the mutant RNF125^C100A/C103A^. Black circles: Zn chelating Cys and His; grey boxes: mutated residues. (**B**) *In vitro* ubiquitination reactions with GST fusion proteins of RNF125 and RNF125^C100A/C103A^ for 60 min at 37 °C. Ubiquitination was detected with anti-ubiquitin (Ub) and input levels of the chimeras with anti-GST Western blotting. (**C**) HEK293T cells transfected with myc-tagged RNF125 or RNF125^C100A/C103A^ were incubated with 100 μg/ml cycloheximide (CHX) for the indicated times. Cell lysates were analyzed by Western blotting with anti-myc (RNF125). Detection of actin was used as loading control. (**D**) HEK293T cells transfected with cDNAs for myc-tagged RNF125 or RNF125^C100A/C103A^ were either left untreated (−) or treated with MG-132 (20 μM) for 2 h. Cell lysates were analyzed by Western blotting with anti-myc (RNF125) and with anti-actin antibodies as loading controls.

To further investigate the role of the C2HC ZnF domain, two of the predicted Zn^2+^ chelating residues, C100 and C103, were mutated to alanines (Figs 1D and 3A). This mutant, RNF125^C100A/C103A^, showed a loss of activity in *in vitro* ubiquitination reactions (Fig. 3B). Importantly, such an inhibition of activity was not observed for proteins with cysteine mutations at either the second or third ZnFs (see Supplementary Fig. S2).

The C2HC ZnF mutant RNF125^C100A/C103A^ also behaved differently from wildtype RNF125 when expressed in HEK293T cells. This mutant had higher steady state levels (Fig. 3C,D) and a longer half-life than wildtype RNF125 (Fig. 3C), suggesting that it underwent lower auto-ubiquitination. Furthermore, expression levels of RNF125^C100A/C103A^ were not greatly affected by proteasome inhibition with MG-132 (Fig. 3D), again in accordance with the mutant being less extensively auto-ubiquitinated. Therefore, we conclude that disrupting the C2HC ZnF structure severely affected ubiquitin ligase activity of RNF125 *in vitro* and in cells, illustrating the essential role of this ZnF domain.

### The C2HC Zn finger and linker are essential for RING-UbcH5a interactions

**Figure 4:**
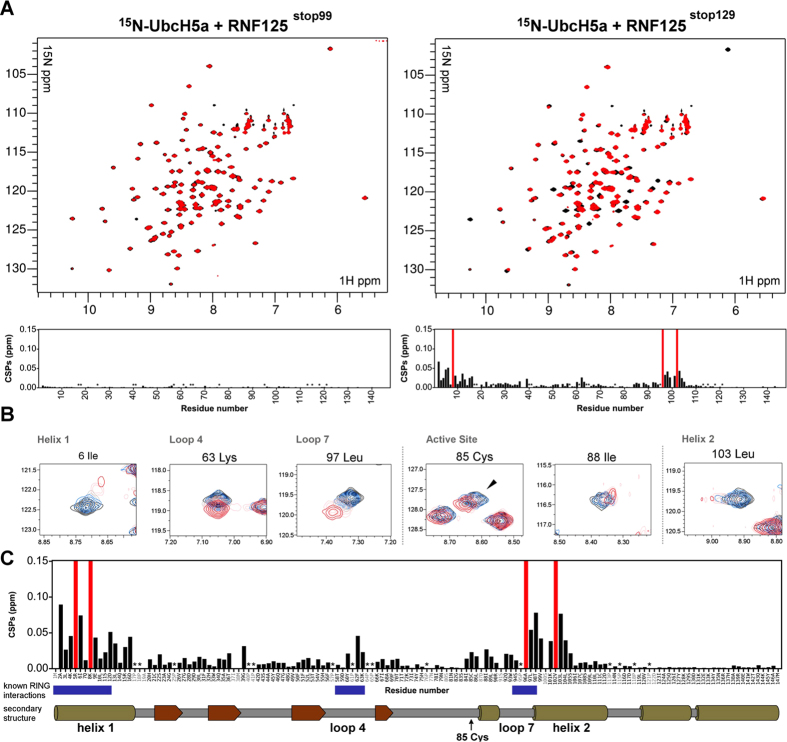
CSPs in ^15^N-UbcH5a caused by either RNF125^stop99^ or RNF125^stop129^. (**A**) Overlay of ^1^H-^15^N HSQC spectra of 0.18 mM ^15^N-UbcH5a before (black), and after (red) addition of 0.5 molar equivalents of RNF125^stop99^ (left) or RNF125^stop129^ (right). Bar graphs represent the combined ^1^H and ^15^N chemical shift perturbations of ^15^N-UbcH5a at these molar ratios. The red bars indicate peaks that have broadened beyond detection. Pro residues are indicated by (*). (**B**) Expanded regions of ^1^H-^15^N HSQC spectra for 0.18 mM UbcH5a showing specific peaks before (black) and after the addition of RNF125^stop129^ at 0.125 (blue), 0.25 (grey), 0.5 (pink), 0.9 (red) molar equivalents. Peaks for residues at known RING binding sites are shown: I6 in helix 1, K63 in loop 4, L97 in loop 7; C85 is the active site Cys; I88 undergoes allosteric changes upon RING binding; L103 is present in helix 2.(**C**) Combined ^1^H and ^15^N chemical shift perturbations of 0.18 mM ^15^N-UbcH5a at 0.9 molar equivalents of RNF125^stop129^. Assigned residues are in black in the sequence underneath the graph, Pro are indicated by a (*). Red bars indicate residues that broadened beyond detection in the presence of RNF125^stop129^. The blue bars below the graph indicate areas that have been published to undergo extensive CSPs in similar NMR titrations: residues 1–12 in helix 1, 58–63 in loop 4 and 94–99 in loop 7. A schematic representation of secondary structure elements (α-helices as cylinders and β-sheets as arrows) of UbcH5a is also shown.

To obtain insights into how the C2HC ZnF (and Li2^120-128^) enhance RNF125 activity, we performed NMR titration experiments that allow a sensitive detection of protein-protein interactions and an identification of the residues involved. For this, ^1^H-^15^N heteronuclear single quantum correlation (HSQC) spectra of the E2 UbcH5a were recorded before and after the addition of either RNF125^stop99^ or RNF125^stop129^. In this way, chemical shift perturbations (CSPs) in ^15^N-UbcH5a caused by either the RING domain alone (RNF125^stop99^), or the RING domain together with the C2HC ZnF plus Li2^120-128^ (RNF125^stop129^) could be distinguished. Strikingly, the results showed that at the same molar ratio of 1:0.5 (^15^N-UbcH5a:RNF125 construct) dramatically more and larger CSPs occurred with RNF125^stop129^ than with RNF125^stop99^ (Fig. 4A). Indeed, some ^15^N-UbcH5a perturbations were already obvious at 0.125 molar equivalents of RNF125^stop129^ (Fig. 4B), whereas those in the presence of 0.5 equivalents of RNF125^stop99^ were still negligible (Fig. 4A). Thus, it can be concluded that the RING domain on its own did not bind to UbcH5a, but that this interaction was greatly enhanced by the presence of the C2HC ZnF and Li2^120-128^.

A backbone assignment of ^13^C^15^N-UbcH5a was performed (see Supplementary Fig. S3) to identify the amino acids that underwent perturbations in the presence of RNF125^stop129^. This revealed that extensive CSPs occurred at well-defined RING binding sites in the UbcH5a alpha helix 1 and loop L7, as well as at loop L4^36^,^37^,^38^,^39^, although the latter ones were of a lesser degree (Fig. 4C, shown for a 1:0.9 ratio of ^15^N-UbcH5a:RNF125^stop129^). Previously described CSPs indicative of allosteric changes induced by RING interactions were also detected, most notably at I88 (Fig. 4B,C). This is believed to be important for the release of ubiquitin at the active site Cys85^40^, where CSPs were observed as well (Fig. 4B,C). Additionally, some large CSPs were observed at UbcH5a regions normally not affected to this extent by the interaction with RING domains. This was most notable for residues at the C-terminus of helix 1 (aa 13-16) and to an even greater extent for amino acids 101-104 in helix 2 (Fig. 4B,C). This suggests that some non-canonical E2 interactions may be occurring as well.

Most importantly, it can be concluded that the C2HC ZnF and Li2^120-128^ are essential for establishing interactions between the RING domain and UbcH5a, hence explaining their role in RNF125 activity.

### Ubch5a causes extensive chemical shift changes in the C2HC Zn finger and Li2^120-128^

To study the RNF125/UbcH5a interaction further, the reciprocal NMR titration experiments were performed, adding unlabeled UbcH5a to ^15^N-RNF125^stop129^. During long NMR experiments (>24 h) spectra changes occurred as a result of partial proteolysis of RNF125^stop129^, which was confirmed by the observation of a lower Mr protein (see Supplementary Fig. S4). These slow changes hindered the NMR assignment of RNF125^stop129^. Therefore, we generated a new protein, RNF125^start31/stop129^, that starts at amino acid 31 (Fig. 1E) and lacks the predicted unstructured 30 N-terminal residues, which indeed resulted in a more stable protein. We determined whether this protein had similar *in vitro* ubiquitination activity as RNF125^stop129^, which was the case (see Supplementary Fig. S4). Furthermore, HSQC spectra of RNF125^stop129^ and RNF125^start31/stop129^ were almost identical apart from the absence of peaks in the central random coil chemical shift area of RNF125^start31/stop129^, consistent with the lack of an unstructured region (see Supplementary Fig. S5). Thus, the absence of the 30 N-terminal amino acids did not affect either the activity or overall structure of the protein in a major way.

**Figure 5:**
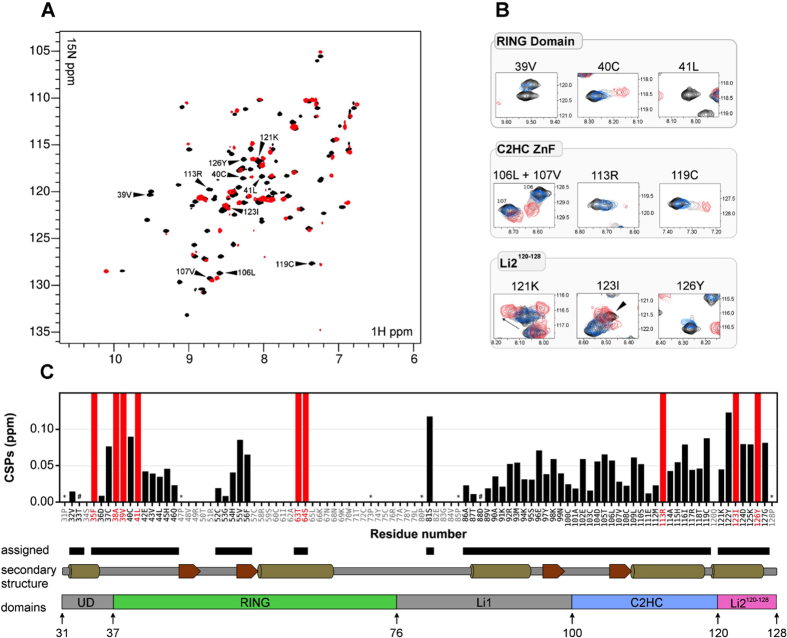
CSPs caused by UbcH5a in ^15^N-RNF125^start31/stop129^. (**A**) Overlay of ^1^H-^15^N HSQC spectra of 0.2 mM ^15^N-RNF125^start31/stop129^ collected at 600 MHz before (black), and after addition of 2 molar equivalents of UbcH5a (red). Specific peaks shown in panel B are indicated by arrows. (**B**) Expanded regions of ^1^H-^15^N HSQC spectra showing specific residues in the RING domain, C2HC ZnF and Li2^120-128^, respectively. Overlays are shown of 0.2 mM ^15^N-RNF125^start31/stop129^ before (black), or after UbcH5a was added at a molar ratio of 0.25 (blue), 0.5 (grey), 1 (pink) or 2 (red). Some peaks (V39, L41, R113) broadened beyond detection at the higher molar ratios of UbcH5a. (**C**) A plot of the combined ^1^H and ^15^N chemical shift perturbations for ^15^N-RNF125^start31/stop129^ residues obtained at 2 molar equivalents of UbcH5a (see **A**). Assigned residues are in black in the sequence underneath the graph, Pro residues are indicated by (*). ^#^indicate T33 and D88 that were too broad in our spectra to be included in the CSP calculation. Red bars represent residues that broadened beyond detection in the presence of UbcH5a. Diagrams of secondary structure elements (α-helices as cylinders, and β-sheets as arrows) and the RNF125^stop129^ domain organization are also shown.

Backbone assignment of RNF125^start31/stop129^ could be achieved for 67% of the peaks, which includes half of the RING domain and the complete C2HC ZnF and Li2^120-128^ regions (Fig. 5, see Supplementary Fig. S6 for more details). The majority of expected peaks could be detected although some where broad, creating assignment gaps. The unassigned peaks cluster in a central region that represents part of the RING domain and linker 1. We observed double peak resonances for residues close to the C-terminus and in the C2HC ZnF, probably arising from the presence of a C-terminal proline in the truncated construct with the capacity to adopt a cis peptide bond^41^ (see Supplementary Fig. S6).

To investigate the effects of E2 binding, HSQC spectra of ^15^N-RNF125^start31/stop129^ were recorded before and after the addition of unlabeled UbcH5a at molar equivalents ranging from 0.125 to 2.5. This resulted in large CSPs or extensive peak broadening at RING residues that were expected to interact with E2s, such as V39, C40 and L41 in the first Zn chelating loop and T63 and S64 in the central alpha helix^9^ (Fig. 5). Peak broadening beyond detection was already observed at 0.25 molar equivalents of UbcH5a for some residues in the RING domain (see Supplementary Fig. S7 for plots of all titration points). In addition, most residues of the C2HC ZnF and Li2^120-128^ also underwent chemical shift changes or peak broadening (Fig. 5B,C, see Supplementary Fig. S7). The most affected regions were the C-terminal residues of the C2HC ZnF and Li2^120-128^. To ascertain that also in these experiments, the absence of the N-terminal region did not influence results, the ^15^N-RNF125^start31/stop129^ CSPs were compared with those obtained in a similar NMR experiment with the longer ^15^N-RNF125^stop129^. This showed that UbcH5a caused near identical perturbations in ^15^N-RNF125^start31/stop129^ and ^15^N-RNF125^stop129^ (Supplementary Fig. S8).

In summary, from the experiments presented so far, we conclude that both the RING and C2HC ZnF domain of RNF125 are needed to form a complex with the E2, and that both domains as well as the Li2^120-128^ sense the formation of the E2-E3 complex.

### X-ray structure of RNF125^stop129^ reveals extensive intramolecular interactions

**Figure 6:**
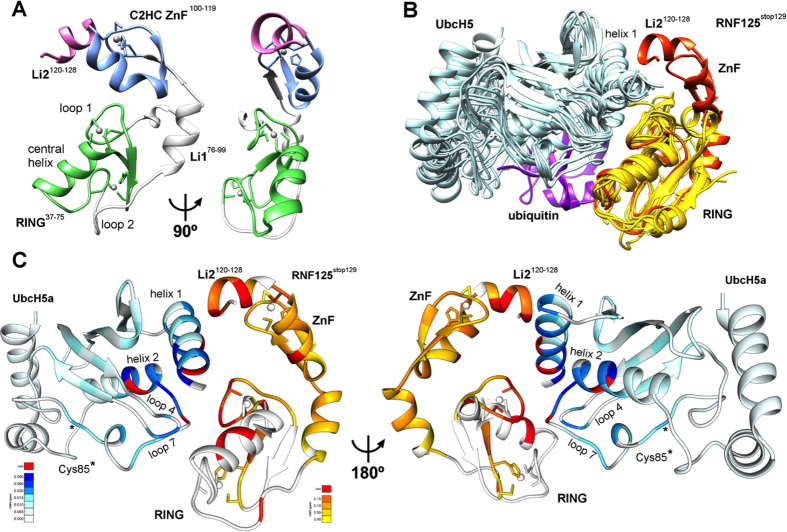
Crystal structure of RNF125^stop129^; models of UbcH5a interactions. (**A**) Ribbon diagram of the RNF125^stop129^ structure with the RING domain in green, the C2HC ZnF in blue, the linker region L1 in grey and Li2^120-128^ in pink. A 90° rotated view is also shown. (**B**) Overlays of the RNF125^stop129^ structure (orange) with eight X-ray structures of RING domains (yellow) co-crystallized with UbcH5 E2 proteins (blue) or with a UbcH5a-Ub conjugate (4AP4). Ubiquitin is in purple. Structures from PDB were: 4QPL (RNF146/UbchH5a); 2YHO (Mylip/UbcH5a); 4V3K (RNF38/UbcH5b); 4AUQ (Birc7/UbcH5b); 4A4C (Cbl/UbcH5b); 3EB6 (IAP/ UbcH5b); 3RPG (Bmi/UbcH5c). Some PDB files were altered to depict the RING domain and E2 only. (C) Model of RNF125^stop129^ in association with UbcH5a, generated by overlaying RNF125^stop129^ with RNF146 in PDB 4QPL. The CSPs observed in the NMR experiments of Fig. 4 (^15^N-UbcH5a/RNF125^start31/stop129^ at 1:0.9) and Fig. 5 (^15^N-RNF125^stop129^/Ubch5a at 1:2) are highlighted on RNF125^stop129^ (yellow-orange) and UbcH5a (cyan-blue). Residues that broadened beyond detection are in red. A 180° rotated view is also shown.

To obtain additional structural insights into the relationship between the RING and C2HC ZnF, we attempted the crystallization of full length RNF125. Unfortunately, this protein was not soluble enough. However, the structure of the truncated RNF125^stop129^ was solved at a resolution of 1.55 Å (Table 1). RNF125^stop129^ shows a compact module in which the RING domain and the C2HC ZnF are easily discerned (Fig. 6A, see Supplementary Fig. S9). The predicted unstructured N-terminal 30 amino acids were not observed in the electron density. As shown previously, this region does not have a major impact on activity and folding of RNF125^stop129^ (see Supplementary Figs S4 and S5). The very low evolutionary sequence conservation of this region also argues against an important structural role (see Supplementary Fig. S10).

The structure of the RING domain of RNF125 resembles that of other RING domains in the PDB database. Overlays with eight RING domains that have been co-crystallized with UbcH5 E2 proteins^5^,^6^,^12^,^14^,^20^,^42^,^43^,^44^ showed that RNF125 contains similar E2 interacting surfaces (Fig. 6B) composed of the two zinc chelating loops and part of the central α-helix. The C2HC ZnF adopts a ββα fold, typical for ZnF domains, which is capped on both sides by α-helices formed by regions Li1 and Li2^120-128^ (Fig. 6A). The α-helix of Li2^120-128^ is almost continuous with that of the C2HC ZnF, but is kinked at C119. As expected, C100, C103, H115 and C119 coordinate the Zn atom (Fig. 6A).

The overlays of RNF125^stop129^ with RING/UbcH5 complexes (Fig. 6B) predict that upon interaction with an E2, the C2HC ZnF/Li2^120-128^ region would be situated close to alpha helix 1 of the E2. A UbcH5a~Ub conjugate was included in the overlay to mark the position of ubiquitin. This showed that the C2HC ZnF/Li2^120-128^ region is unlikely to contact a ubiquitin that is bound to an E2 in the closed conformation (Fig. 6B).

We mapped the CSPs of Figs 4 and 5 onto a model of a RNF125^stop129^/UbcH5a complex, made by overlaying the RNF125^stop129^ structure with that of RNF146 in a Ubch5a co-crystal^20^ (Fig. 6C). This showed that, as expected, large CSPs can be observed in both RNF125 and UbcH5a at the predicted RING-E2 interface. Outside the RING domain of RNF125^stop129^, the largest CSPs occur at the C-terminus of Li2^120-128^. This region faces towards the C-terminal part of UbcH5a alpha helix 1 (Fig. 6D), where UbcH5a undergoes non-canonical CSPs (at residues 13–16). Other non-canonical UbcH5a CSPs at residues 101–104 in helix 2 may have been propagated from here (Fig. 6E). This may suggest that Li2^120-128^ makes direct contacts with the E2, but this remains to be established directly.

**Figure 7:**
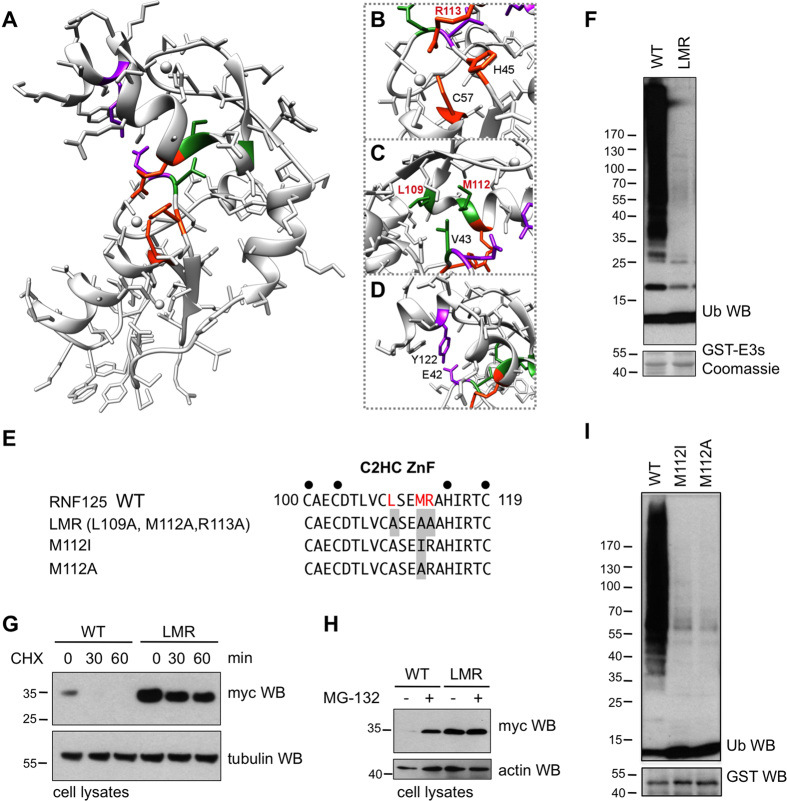
Effects of mutations at C2HC ZnF residues that contact the RING domain. (**A**) RNF125^stop129^ structure with side chains showing. Residues involved in intramolecular interactions, also shown in (**B**–**D**), are highlighted. (**B**) Residues involved in electrostatic contacts between the C2HC ZnF and RING are in orange. R113 (in red) was mutated to Ala in the RNF125^LMR^ mutant (panels **E**–**H**). (**C**) Residues involved in hydrophobic contacts between the C2HC ZnF and RING are in green. Residues in red, L109 and M112, were mutated (panels **E**–**I**). (**D**) Residues involved in hydrophobic contacts between the Li2^120-128^ and RING are in magenta. (**E**) Amino acid sequence of the C2HC ZnF for RNF125 and the mutants used in panels (**E–I**). Black circles: Zn chelating Cys and His; grey boxes: mutated residues. (**F**) *In vitro* ubiquitination reactions with GST fusion proteins of RNF125 and RNF125^L109/M112/R113A^ (LMR) for 60 min at 37 °C. Ubiquitination was detected with anti-ubiquitin (Ub) and input levels of the chimeras with Coomassie staining of separately loaded equivalent amounts of protein. (**G**) HEK293T cells transfected with myc-tagged RNF125 or RNF125^L109A,M112A,R113A^ (LMR) were incubated with 100 μg/ml cycloheximide (CHX) for the indicated times. Cell lysates were analyzed by Western blotting with anti-myc (RNF125). Detection of tubulin was used as loading control. (**H**) HEK293T cells transfected with cDNAs for myc-tagged RNF125 or RNF125^L109A,M112A,R113A^ (LMR) were either left untreated (-) or treated with MG-132 (20 μM) for 2 h. Cell lysates were analyzed by Western blotting with anti-myc (RNF125) and with anti-actin antibodies as loading controls. (**I**) *In vitro* ubiquitination reactions with GST fusion proteins of RNF125 and the mutants RNF125^M112I^ and RNF125^M112A^, for 60 min at 37 °C. Ubiquitination was detected with anti-ubiquitin (Ub) and input levels of the chimeras with anti-GST.

Interestingly, the structure demonstrates that extensive intramolecular interactions take place between the RING domain and the C2HC ZnF (Fig. 7A). Specifically, electrostatic interactions can be observed between C57 coordinating the proximal Zn^2+^ of the RING domain and R113 in the C2HC ZnF, where R113 makes additional hydrophobic contacts with H45 in the RING domain (Fig. 7B). Furthermore, a hydrophobic patch is formed between V43 in the RING, and L109/M112 in the C2HC ZnF (Fig. 7C). Additionally, an anchor between the RING and Li2^120-128^ is provided by a strong hydrogen bond (2.6 Å) between E42 O_ε2_ and the hydroxyl group of Tyr122 (Fig. 7D). These extensive interactions provide a structural basis for the essential role of the C2HC ZnF in RNF125 activity, and are consistent with the concept that the RING and C2HC ZnF together form a functional unit required for binding to the E2.

### Mutations at RING contact sites in the C2HC ZnF abolish RNF125 activity

To test the prediction that the C2HC ZnF is important for the RING structure, we measured the HSQC spectrum of the RING-only construct ^15^N-RNF125^stop99^, as well as that of a shorter form that lacks the N-terminal region, ^15^N-RNF125^start31/stop99^. The spectra of both proteins show low spectral dispersion and non-uniform peak intensities, typical for misfolded and aggregated proteins (see Supplementary Fig. S11). Thus, in the absence of the C2HC ZnF and Li2^120-128^ a properly folded RING domain could not be obtained.

To investigate the structural role of the C2HC ZnF further, Ala substitutions were introduced at residues that, based on the crystal structure, interact with residues in the RING domain. For this, ZnF residues L109, M112 and R113 (Fig. 7B,C) were mutated simultaneously in the full length protein (Fig. 7E). Strikingly, the resulting mutant, RNF125^LMR^ showed an almost complete lack of *in vitro* ubiquitin ligase activity (Fig. 7F). These ZnF mutations were also introduced into a construct for expression in mammalian cells. In accordance with reduced auto-ubiquitination activity, RNF125^L109/M112/R113A^ showed higher steady state levels (Fig. 7G,H), a longer half life (Fig. 7G) and less extensive targeting to the proteasome (Fig. 7H) than wild type RNF125. Next, we focused on one of these residues, M112, which is highly conserved (see Supplementary Fig. S10) and reported to be associated with an overgrowth syndrome characterized by developmental defects and inflammatory disease^31^. Thus, M112 was mutated to an Ala, and to an Ile (Fig. 7E) to generate the RNF125 mutant detected in some of the overgrowth syndrome patients^31^. Strikingly, both single M112 mutations resulted in a drastic reduction in ubiquitin ligase activity of RNF125 (Fig. 7I). Thus, we conclude that the activity of RNF125 is absolutely dependent on both the RING and the ZnF domain and that the disruption of this functional unit by a single point mutation is sufficient to inhibit activity.

## Discussion

The large number and heterogeneity of RING ubiquitin ligases suggests that also many different regulatory mechanisms for these proteins exist. Indeed, we have shown that even related proteins, such as RNF125 and RNF114, can have extensive differences in activity. Analyzing such differences may lead to important new insights into requirements for activity, which was the premise for the current investigation. Using mutational analysis, activity based assays, NMR and X-ray crystallography, we have demonstrated the essential role of a C2HC ZnF for the interaction of RNF125 with the E2, which originates from the requirement of the C2HC ZnF for the structural stability of the RING domain.

At the outset, the generation of RNF125/RNF114 chimeras demonstrated that the RING domain of RNF125 is not sufficient for activity, which was confirmed with C-terminally truncated stop mutants (Fig. 2). Additional constructs showed that the presence of the C2HC ZnF is required, and that mutations at Zn^2+^ chelating Cys of this domain resulted in a loss of activity (Fig. 3). In addition, linker 2 residues 120–128, Li2^120-128^, were found to contribute to activity in a non-essential way (Fig. 2). The loss of activity for a truncated RNF125 protein that contains the RING but not the C2HC ZnF/Li2^120-128^ region, correlated with a loss of interaction with ^15^N-UbcH5a seen by NMR (Fig. 4). The crystal structure of the stop mutant RNF125^stop129^ showed clear contacts between the RING and C2HC ZnF domains (Fig. 6). The same construct showed sharp signals by NMR for most of the RING and C2HC ZnF domains (Fig. 6), although the presence of some broad lines suggests that the connecting segment may be dynamic.

We hypothesized that the presence of the C2HC ZnF domain could be required for the stability of the RING domain. This was confirmed with a construct containing the RING domain sequence but lacking the C2HC ZnF/Li2^120-128^ region, which was found to be unfolded (see Supplementary Fig. S11). Moreover, the essential role of the RING-C2HC ZnF interaction was also established by mutations at residues L109, R113 and M112 in the ZnF that are in direct contact with the RING domain. The triple mutant showed a loss of ubiquitin ligase activity *in vitro* and in cells (Fig. 7). Strikingly, of these three RING interacting residues, only the M112 equivalent is not present in RNF114 but is an Ile instead (see Supplementary Fig. S1). Moreover, a M112 to I112 mutation was identified in patients with the recently described Tenorio overgrowth syndrome^31^. This M112 is highly conserved throughout evolution, also suggesting an important role. A key role for M112 was confirmed with single M112A and M112I mutations that showed a severe inhibition of RNF125 *in vitro* ubiquitin ligase activity (Fig. 7). Collectively, our experiments show that the RING and C2HC ZnF domains with their linker regions act as a functional unit held together by interdomain contacts.

Thus, the primary role of the C2HC ZnF seems to be the stabilization of the RING domain and the Li^120-128^ region is likely to support this role. However, the question arises whether the C2HC ZnF/Li^120-128^ region also participates directly in an interaction with the E2. The addition of UbcH5a to ^15^N-RNF125^stop129^, caused CSPs in the ZnF/Li2^120-128^ region as well as in the RING domain (Fig. 5, see Supplementary Fig. S7). Since these CSPs include both exposed and buried residues, some of these may be caused by a rearrangement of the RING-C2HC ZnF interaction in an E2 complex. In the reciprocal NMR experiment, CSPs induced in ^15^N-UbcH5a were predominantly those expected from canonical RING binding sites, but some large non-canonical CSPs were observed as well, most notably at residues 13–16 in UbcH5a helix 1, and 101–104 in helix 2 (Fig. 4). An overlay of structures for RNF125^stop129^ and a RNF146/UbcH5a complex showed that the C2HC ZnF+Li2^120-128^ region would be in the correct location for an interaction with the C-terminus of UbcH5a helix 1 (aa 13-16), from where allosteric changes could be propagated to residues 101–104 (Fig. 6). This is an interesting possibility that warrants future exploration by NMR and crystallography with E2-Ub conjugates, especially since K101 and L104 interact with ubiquitin in the “closed” conformation, which is important for ubiquitin release^5^,^6^,^7^. Moreover, an interaction with the C-terminal region of UbcH5 helix 1, would be expected to compete with E1 binding to the E2^10^. At present we do not exclude secondary contacts between ZnF/Li2^120-128^ and the E2, but we have no direct evidence for this.

**Figure 8:**
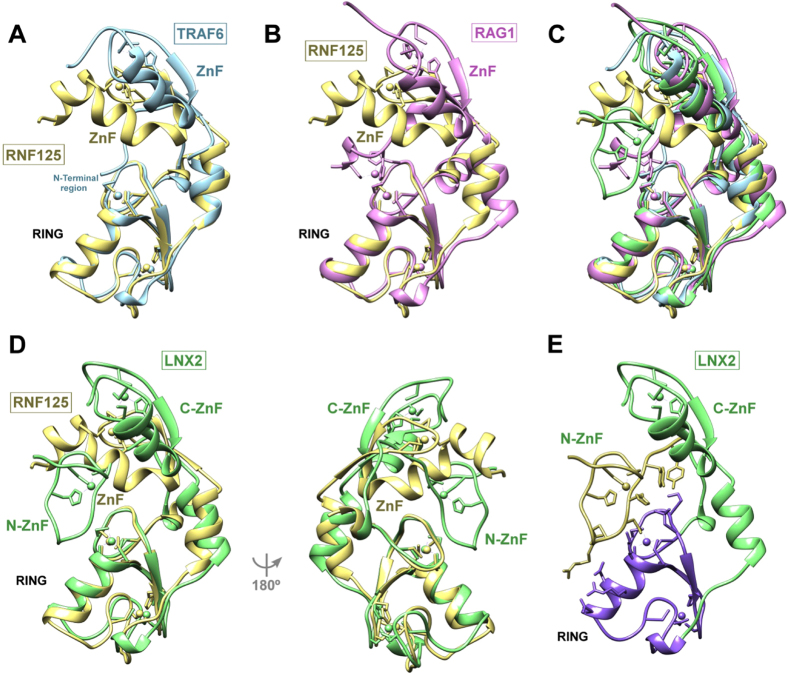
Comparison of the RNF125 structure with that of TRAF6, RAG1 and LNX2. (**A**) Overlay of the RNF125^stop129^ structure (yellow) with that of TRAF6 RING and ZnF domains (blue) in PDB 3HCU. (**B**) Overlay of the RNF125^stop129^ structure (yellow) with that of RAG1 RING and ZnF domains (pink) in PDB 1RMD. (**C**) Overlay of RNF125^stop129^ with TRAF6, RAG1 and LNX2. Colours and PDBs as in (**A**,**B**,**D**). (**D**) Overlay of the RNF125^stop129^ structure (yellow) with that of LNX2 RING and ZnF domains (green) in PDB 5DIN. Two orientations are shown. N-ZnF: N-terminal ZnF; C-ZnF: C-terminal ZnF. (**E**) LNX2 structure with side chains shown for residues that interact at the N-ZnF/RING interface. The N-ZnF and RING are in yellow and purple, respectively.

The X-ray structure of RNF125^stop129^ resembles that of three other partial protein structures with adjacent RING and ZnF domains, TRAF6^45^, RAG1^46^ and LNX2^47^. Significantly, the ZnF1 of TRAF6 is also essential for E2 interactions, but the mechanism for this is drastically different from that of RNF125. The ZnF1 of TRAF6 has no direct effect on the RING domain but fixes the position of an N-terminal region that makes contacts with the E2^45^. TRAF6 does not show the extensive contacts between the RING and ZnF as seen in RNF125 (Fig. 8A), and its ZnF1 is oriented away from the E2. In the case of RAG1, the ZnF is in a similar position to that of TRAF6 (Fig. 8B,C), and together with the RING was found to be involved in dimerization^46^. However, for RNF125 we did not detect dimerization in relaxation experiments (see Supplementary Fig. S12). Recently the structure of LNX2, a ubiquitin ligase that contains a ZnF on both sides of the RING domain, was solved^47^. As for RNF125, one of the ZnFs was found to be essential for activity as well as for solubility of the RING domain. However, this ZnF is located to the N-terminus of the RING and does not adopt a typical ZnF fold. Nevertheless, it is located in a similar position to the RNF125 C2HC ZnF (Fig. 8D) and also makes extensive contacts with the first Zinc binding loop of the RING domain (Fig. 8E). The C-terminal ZnF of LNX2, on the other hand, is located in a similar position as those of TRAF6 and RAG1 (Fig. 8C). Thus, although distinct in primary and secondary structure, the ZnFs of RNF125 and LNX2 seems to perform a comparable structural role.

Despite the differences between these proteins, an important role of the ZnFs flanking the RING domain appears as a common element. It can be envisioned that these ZnF domains play a role in regulating ubiquitin ligase activities through interactions with ligands (proteins or nucleic acids), metal chelating molecules or post translational modifications affecting the contacts, and therefore the stability and activity of the RING domain. We are currently exploring these possibilities.

## Methods

### Reagents

Reagents were from Sigma-Aldrich unless otherwise stated.

### Plasmids

All cDNAs were of human origin. For expression of N-terminal GST fusion proteins in *Escherichia Coli*, cDNAs for RNF125, RNF114, UbcH5a were cloned into pGEX4T3 (GE Healthcare). cDNAs for RNF125 and RNF114 in pcDNA3.1myc/HisA- (Invitrogen), and HA-Ubiquitin in pcDNA3.1 for expression in mammalian cells have been previously described^32^,^33^. Point mutations, including those that introduce stop codons, were generated using Quickchange lightning (Agilent technologies). cDNAs for RNF125/RNF114 chimeras were made by overlapping PCRs and subsequently subcloned into pGEX4T3 and pcDNA3.1myc/HisA-. All constructs were verified by DNA sequencing.

### Expression and purification of GST fusion proteins

GST-fusion proteins were expressed in *Escherichia Coli* BL21DE3(pLysS) (Novagen), induced with 1 mM isopropyl β-D-1-thiogalactopyranoside (IPTG, Duchefa) and purified using glutathione-sepharose beads (GE healthcare) according to the manufacturer’s instructions. Bacterial cell pellets were resuspended in 50 mM Tris pH 7.4, 150 mM NaCl, 200 μM ZnCl_2_, 1 mM DTT, supplemented with protease inhibitors (protease complete inhibitor cocktail EDTA-free, GE Healthcare) and disrupted by sonication. The homogenate was centrifuged at 40,000 × g and glutathione-sepharose beads were added to the supernatant. For ubiquitination reactions, cultures of 50 ml were grown in LB medium, IPTG induction was for 2 h at 37 °C and the GST-fusion proteins were left on the glutathione-sepharose beads.

For X-ray crystallography and NMR, proteins were purified from 2 L cultures and induced for 16 h at 20 °C. Cultures for NMR were grown in minimal medium (M9) supplemented with ^15^NH_4_Cl (Cambridge Isotope Laboratories) for HSQC recording, or with both ^15^NH4Cl and ^13^C6-D-glucose (Cambridge Isotope Laboratories) for backbone assignment. For these applications, the proteins were cleaved from GST with thrombin (Sigma) on the beads and thrombin was subsequently removed with benzaminidine-agarose beads (GE healthcare). Following another incubation with glutathione-sepharose beads to remove traces of free GST, the proteins were concentrated to the desired concentration (Centricon 3 K, Millipore). Protein concentrations were determined by Bradford (BioRad) and Nanodrop OD280 reading. Protein purity was monitored by Coomassie gel staining.

### Antibodies

Mouse anti-ubiquitin P4D1 and rabbit anti-GST Z-5 were from Santa Cruz. The mouse monoclonal (mAb) antibody anti-myc 9E10, and anti-tubulin TUB2.1 were from Sigma, mAb anti-P23 JJ3 from Abcam, mAb anti-actin C4 from Millipore and mAb anti-HA HA.11 from Covance. Horse-radish peroxidase-conjugated donkey anti-rabbit (GE healthcare) and goat anti-mouse antibodies (Thermoscientific) were used as secondary antibodies.

### *In vitro* ubiquitination assays

Standard ubiquitination reactions were performed for 60 min at 37 **°**C in a total volume of 50 μl, containing 2 μg E3s (as GST-fusion proteins bound to glutathione-beads), 50 ng E1 (Enzo Life Sciences), 1 μg E2 (Enzo Life Sciences), 2 μg ubiquitin (BostonBiochem) in 50 mM Tris, pH 7.4; 150 mM NaCl; 0.5 mM dithiothreitol (DTT); 10 mM MgATP. For time-course experiments, reactions were started by the addition of ATP and stopped by the addition of 5× reducing Laemmli loading buffer (100 mM DTT final concentration) and boiling at 95 **°**C for 5 min. To detect ubiquitination, reactions were separated on either a 10% acrylamide gel or a 4–12% NuPAGE (Invitrogen) and transferred to PVDF membrane for Western blotting with anti-ubiquitin antibodies.

### Cell culture, transfections, inhibitor treatment and analysis

Human embryonic kidney 293T cells (HEK293T) were grown in DMEM, supplemented with 10% FCS, 100 U/ml penicillin, 0.1 mg/ml streptomycin. HEK293T cells were transfected using polyethylenimine (PEI) (Polysciences) with 3 μg DNA per well of a 6 well plate. Twenty four hours after transfections, cell were either lysed directly or after incubation with 100 μg/ml cycloheximide or 20 μM MG-132 (Santa Cruz Biotech) as indicated. Cells were lysed in NP-40 buffer (1% NonidetP-40, 20 mM Tris pH 7.6, 150 mM NaCl), supplemented with protease inhibitors (GE Healthcare), and left on ice for 30 min. Nuclei and cell debris were removed by centrifugation at 13,000 × g for 5 min at 4 °C. Cell lysates were denatured in reducing Laemmli sample buffer for 5 min. at 95 °C, separated on 10% SDS-polyacrylamide gels and transferred to PVDF for Western blotting.

### NMR spectroscopy, chemical shift perturbations, backbone assignment

^1^H-^15^N-HSQC spectra of ^15^N-labeled samples were recorded in 50 mM Tris pH 7.4, 150 mM NaCl, 200 μM ZnCl_2_, 1 mM DTT, 10% D_2_O at 25 °C with a Bruker 600 MHz Advance III spectrometer equipped with a TCI CryoProbe (Unitat de RMN, Universitat de Barcelona). NMR spectra were processed using Bruker TopSpin 3.0 and analyzed using CcpNmr^48^. Combined chemical shift differences were calculated using the following equation:

CSP = 
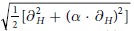
 in which α is 0.14, but 0.2 for Gly^49^. For backbone assignment of ^13^C^15^N-RNF125^start31/stop129^ in 50 mM Tris pH 7.0, 50 mM NaCl, 200 μM ZnCl_2_, 1 mM DTT, a set of standard triple resonance experiments for the sequential assignment was recorded using incremental non-uniform sampling^50^ on Bruker 800 MHz Avance III HD spectrometer equipped with 5 mm TCI CryoProbe (Swedish NMR Centre) at 25 °C. For ^13^C^15^N-UbcH5a assignment in 50 mM Tris pH 7.0, 150 mM NaCl, 200 μM ZnCl_2_, 1 mM DTT, recording was performed with a Bruker 900 MHz Avance III HD spectrometer equipped with 5 mm TCI CryoProbe (Swedish NMR Centre) at 25 °C. Spectra were processed using MDDNMR^51^ and the analysis was performed in CcpNmr Analysis.

### X-ray crystallography

A full sparse vapour diffusion matrix screen was performed at 18 °C on a sample of RNF125^stop129^ at 952 μM in 20 mM TRIS buffer pH 7.4 and 137 mM NaCl, using the Phoenix robot (Art Robbinson Instruments). Crystals from the following conditions were tested for diffraction: 0.2 M KNO3, 20% PEG 3350; 0.1 M HEPES pH 7.5, 10% PEG 8 K; 0.1 M HEPES pH 7.5, 10% PEG 6 K, 5% MPD; 0.1 M TRIS pH 8.5, 20% ethanol; 0.2 M CaAc2, 0.1 M Cacodylate pH 6.5, 40% PEG 300; 0.1 M PCB buffer (PACT screen, Molecular Dimensions, http://www.moleculardimensions.com/), 25% PEG 1500. In addition, crystals were obtained using a batch method by storing the protein sample at 454 μM in phosphate buffer saline (Sigma Aldrich) at 4 °C. The crystals belonged to space group *P*4_3_2_1_2 or *P*2_1_2_1_2_1_. The highest resolution obtained was 1.55 Å (see Table 1) for crystals belonging to space group *P*2_1_2_1_2_1_. The data was measured at the XALOC beamline (synchrotron ALBA, Spain)^38^ and processed using the XDS package^52^ and the structure was solved by molecular replacement using the PHASER program^53^ from the CCP4 software package^54^. Structure refinement was done using REFMAC 5.8.0073^55^ and manually adjusted using Coot 0.7.2^56^. The final R_cryst_ /R_free_ was 21.2% and 24.4%, respectively. The atomic coordinates and the structure factors were deposited in the PDB (reference code 5DKA).

Molecular images were generated using UCSF Chimera^57^.

## Additional Information

**How to cite this article**: Bijlmakers, M.-J. *et al.* A C2HC zinc finger is essential for the RING-E2 interaction of the ubiquitin ligase RNF125. *Sci. Rep.* **6**, 29232; doi: 10.1038/srep29232 (2016).

## Supplementary Material

Supplementary Information

## Figures and Tables

**Table 1:** Data collection and refinement statistics.

	**Rnf125**^**stop129**^
Data collection	
Space group	*P*2_1_2_1_2_1_
Cell dimensions	
*a, b, c* (Å)	51.67, 52.32, 73.97
α, β, γ (°)	90, 90, 90
Resolution (Å)^a^	50-1.55 (1.59-1.55)
*R*_sym_^b^ or *R*_merge_	5.5 (80)
*I*/σ*I*	10.2 (1.3)
Completeness (%)	94.0 (94.7)
Redundancy	2.9 (2.9)
Refinement	
Resolution (Å)	50-1.55
No. reflections	28010 (2192)
*R*_work_^c^/*R*_free_^d^	20.9/23.9
No. atoms	
Protein	1512
Ligand/ion	11
Water	132
*B*-factors	
Protein	16.8
Ligand/ion	37.8
Water	36.0
R.m.s. deviations	
Bond lengths (Å)	0.021
Bond angles (°)	2.14

^a^Throughout the table, the values in parentheses are for the outermost resolution shell.

^b^*R*_sym_ = Σ_h,i_|*Î*_h_ − *I*_h,i_|/Σ_h_ Σ_i_ *I*_h,i_ , where *Î*_h_ = (1/n_h_) Σ_i_ *I*_h,i_ and n_h_ is the number of times a reflection is measured.

^c^*R*_work_ = Σ_hkl_||*F*_obs_| − *k* |*F*_calc_||/Σ_hkl_ |*F*_obs_|.

^d^*R*_free_ = Σ_hkl⊂T_||*F*_obs_| − *k*|*F*_calc_||/Σ_hkl⊂T_ |*F*_obs_| where T represents a test set comprising ~5% of all reflections excluded during refinement.

## References

[b1] Komander D. & Rape M. The ubiquitin code. Annu. Rev. Biochem. 81, 203–229 (2012). 22524316 10.1146/annurev-biochem-060310-170328

[b2] Pickart C. M. & Eddins M. J. Ubiquitin: structures, functions, mechanisms. Biochim. Biophys. Acta. 1695, 55–72 (2004). 15571809 10.1016/j.bbamcr.2004.09.019

[b3] Berndsen C. E. & Wolberger C. New insights into ubiquitin E3 ligase mechanism. Nat. Struct. Mol. Biol. 21, 301–307 (2014). 24699078 10.1038/nsmb.2780

[b4] Deshaies R. J. & Joazeiro C. A. P. RING domain E3 ubiquitin ligases. Annu. Rev. Biochem. 78, 399–434 (2009). 19489725 10.1146/annurev.biochem.78.101807.093809

[b5] Dou H., Buetow L., Sibbet G. J., Cameron K. & Huang D. T. BIRC7-E2 ubiquitin conjugate structure reveals the mechanism of ubiquitin transfer by a RING dimer. Nat. Struct. Mol. Biol. 19, 876–883 (2012). 22902369 10.1038/nsmb.2379 PMC3880866

[b6] Plechanovová A., Jaffray E. G., Tatham M. H., Naismith J. H. & Hay R. T. Structure of a RING E3 ligase and ubiquitin-loaded E2 primed for catalysis. Nature 489, 115–120 (2012). 22842904 10.1038/nature11376 PMC3442243

[b7] Pruneda J. N. *et al.* Structure of an E3:E2~Ub complex reveals an allosteric mechanism shared among RING/U-box ligases. Mol. Cell 47, 933–942 (2012). 22885007 10.1016/j.molcel.2012.07.001 PMC3462262

[b8] Budhidarmo R., Nakatani Y. & Day C. L. RINGs hold the key to ubiquitin transfer. Trends Biochem. Sci. 37, 58–65 (2012). 22154517 10.1016/j.tibs.2011.11.001

[b9] Metzger M. B., Pruneda J. N., Klevit R. E. & Weissman A. M. RING-type E3 ligases: master manipulators of E2 ubiquitin-conjugating enzymes and ubiquitination. Biochim. Biophys. Acta. 1843, 47–60 (2014). 23747565 10.1016/j.bbamcr.2013.05.026 PMC4109693

[b10] Stewart M. D., Ritterhoff T., Klevit R. E. & Brzovic P. S. E2 enzymes: more than just middle men. Cell Res. 26, 423–440 (2016). 27002219 10.1038/cr.2016.35 PMC4822130

[b11] Vittal V., Stewart M. D., Brzovic P. S. & Klevit R. E. Regulating the Regulators: Recent Revelations in the Control of E3 Ubiquitin Ligases. J. Biol. Chem. 290, 21244–21251 (2015). 26187467 10.1074/jbc.R115.675165 PMC4571856

[b12] Mace P. D. *et al.* Structures of the cIAP2 RING domain reveal conformational changes associated with ubiquitin-conjugating enzyme (E2) recruitment. J. Biol. Chem. 283, 31633–31640 (2008). 18784070 10.1074/jbc.M804753200

[b13] Liew C. W., Sun H., Hunter T. & Day C. L. RING domain dimerization is essential for RNF4 function. Biochem. J. 431, 23–29 (2010). 20681948 10.1042/BJ20100957 PMC3104014

[b14] Zhang L. *et al.* The IDOL-UBE2D complex mediates sterol-dependent degradation of the LDL receptor. Genes Dev. 25, 1262–1274 (2011). 21685362 10.1101/gad.2056211 PMC3127428

[b15] Linke K. *et al.* Structure of the MDM2/MDMX RING domain heterodimer reveals dimerization is required for their ubiquitylation in trans. Cell Death Differ. 15, 841–848 (2008). 18219319 10.1038/sj.cdd.4402309

[b16] Brzovic P. S., Rajagopal P., Hoyt D. W., King M. C. & Klevit R. E. Structure of a BRCA1-BARD1 heterodimeric RING-RING complex. Nat. Struct. Biol. 8, 833–837 (2001). 11573085 10.1038/nsb1001-833

[b17] Li Z. *et al.* Structure of a Bmi-1-Ring1B polycomb group ubiquitin ligase complex. J. Biol. Chem. 281, 20643–20649 (2006). 16714294 10.1074/jbc.M602461200

[b18] Buchwald G. *et al.* Structure and E3-ligase activity of the Ring-Ring complex of polycomb proteins Bmi1 and Ring1b. EMBO J. 25, 2465–2474 (2006). 16710298 10.1038/sj.emboj.7601144 PMC1478191

[b19] Alvarez S. E. *et al.* Sphingosine-1-phosphate is a missing cofactor for the E3 ubiquitin ligase TRAF2. Nature 465, 1084–1088 (2010). 20577214 10.1038/nature09128 PMC2946785

[b20] DaRosa P. A. *et al.* Allosteric activation of the RNF146 ubiquitin ligase by a poly(ADP-ribosyl)ation signal. Nature 517, 223–226 (2015). 25327252 10.1038/nature13826 PMC4289021

[b21] Das R. *et al.* Allosteric activation of E2-RING finger-mediated ubiquitylation by a structurally defined specific E2-binding region of gp78. Mol. Cell 34, 674–685 (2009). 19560420 10.1016/j.molcel.2009.05.010 PMC3050579

[b22] Das R. *et al.* Allosteric regulation of E2:E3 interactions promote a processive ubiquitination machine. EMBO J. 32, 2504–2516 (2013). 23942235 10.1038/emboj.2013.174 PMC3770950

[b23] Li S. *et al.* Insights into Ubiquitination from the Unique Clamp-like Binding of the RING E3 AO7 to the E2 UbcH5B. J. Biol. Chem. 290, 30225–30239 (2015). 26475854 10.1074/jbc.M115.685867 PMC4683248

[b24] Dou H., Buetow L., Sibbet G. J., Cameron K. & Huang D. T. Essentiality of a non-RING element in priming donor ubiquitin for catalysis by a monomeric E3. Nat. Struct. Mol. Biol. 20, 982–986 (2013). 23851457 10.1038/nsmb.2621 PMC4471106

[b25] Chu P. *et al.* Systematic identification of regulatory proteins critical for T-cell activation. J. Biol. 2, 21 (2003). 12974981 10.1186/1475-4924-2-21 PMC333404

[b26] Arimoto K.-I. *et al.* Negative regulation of the RIG-I signaling by the ubiquitin ligase RNF125. Proc. Natl. Acad. Sci. USA 104, 7500–7505 (2007). 17460044 10.1073/pnas.0611551104 PMC1863485

[b27] Shoji-Kawata S. *et al.* The RING finger ubiquitin ligase RNF125/TRAC-1 down-modulates HIV-1 replication in primary human peripheral blood mononuclear cells. Virology 368, 191–204 (2007). 17643463 10.1016/j.virol.2007.06.028

[b28] Yang L. *et al.* RNF125 is a ubiquitin-protein ligase that promotes p53 degradation. Cell. Physiol. Biochem. 35, 237–245 (2015). 25591766 10.1159/000369691

[b29] Britto A. M. A. *et al.* Expression levels of the innate response gene RIG-I and its regulators RNF125 and TRIM25 in HIV-1-infected adult and pediatric individuals. AIDS 27, 1879–1885 (2013). 24131985 10.1097/QAD.0b013e328361cfbf

[b30] Kim H. *et al.* Downregulation of the Ubiquitin Ligase RNF125 Underlies Resistance of Melanoma Cells to BRAF Inhibitors via JAK1 Deregulation. Cell Rep. 11, 1458–1473 (2015). 26027934 10.1016/j.celrep.2015.04.049 PMC4681438

[b31] Tenorio J. *et al.* A new overgrowth syndrome is due to mutations in RNF125. Hum. Mutat. 35, 1436–1441 (2014). 25196541 10.1002/humu.22689

[b32] Giannini A. L., Gao Y. & Bijlmakers M.-J. T-cell regulator RNF125/TRAC-1 belongs to a novel family of ubiquitin ligases with zinc fingers and a ubiquitin-binding domain. Biochem. J. 410, 101–111 (2008). 17990982 10.1042/BJ20070995 PMC2733222

[b33] Bijlmakers M.-J., Kanneganti S. K., Barker J. N., Trembath R. C. & Capon F. Functional analysis of the RNF114 psoriasis susceptibility gene implicates innate immune responses to double-stranded RNA in disease pathogenesis. Hum. Mol. Genet. 20, 3129–3137 (2011). 21571784 10.1093/hmg/ddr215 PMC3140818

[b34] Capon F. *et al.* Identification of ZNF313/RNF114 as a novel psoriasis susceptibility gene. Hum. Mol. Genet. 17, 1938–1945 (2008). 18364390 10.1093/hmg/ddn091 PMC2900900

[b35] Zhao H. *et al.* A novel E3 ubiquitin ligase TRAC-1 positively regulates T cell activation. J. Immunol. 174, 5288–5297 (2005). 15843525 10.4049/jimmunol.174.9.5288

[b36] Benirschke R. C. *et al.* Molecular basis for the association of human E4B U box ubiquitin ligase with E2-conjugating enzymes UbcH5c and Ubc4. Structure 18, 955–965 (2010). 20696396 10.1016/j.str.2010.04.017 PMC3005147

[b37] Dominguez C. *et al.* Structural Model of the UbcH5B/CNOT4 Complex Revealed by Combining NMR, Mutagenesis, and Docking Approaches. Structure 12, 633–644 (2004). 15062086 10.1016/j.str.2004.03.004

[b38] Nordquist K. A. *et al.* Structural and functional characterization of the monomeric U-box domain from E4B. Biochemistry 49, 347–355 (2010). 20017557 10.1021/bi901620v PMC2806929

[b39] van Wijk S. J. L. & Timmers H. T. M. The family of ubiquitin-conjugating enzymes (E2s): deciding between life and death of proteins. The FASEB Journal 24, 981–993 (2010). 19940261 10.1096/fj.09-136259

[b40] Ozkan E., Yu H. & Deisenhofer J. Mechanistic insight into the allosteric activation of a ubiquitin-conjugating enzyme by RING-type ubiquitin ligases. Proc. Natl. Acad. Sci. USA 102, 18890–18895 (2005). 16365295 10.1073/pnas.0509418102 PMC1316884

[b41] Bernstein B. E., Hoffman R. C., Horvath S., Herriott J. R. & Klevit R. E. Structure of a histidine-X4-histidine zinc finger domain: insights into ADR1-UAS1 protein-DNA recognition. Biochemistry 33, 4460–4470 (1994). 8161501 10.1021/bi00181a005

[b42] Buetow L. *et al.* Activation of a primed RING E3-E2-ubiquitin complex by non-covalent ubiquitin. Mol. Cell 58, 297–310 (2015). 25801170 10.1016/j.molcel.2015.02.017

[b43] Dou H. *et al.* Structural basis for autoinhibition and phosphorylation-dependent activation of c-Cbl. Nat. Struct. Mol. Biol. 19, 184–192 (2012). 22266821 10.1038/nsmb.2231 PMC3880865

[b44] Bentley M. L. *et al.* Recognition of UbcH5c and the nucleosome by the Bmi1/Ring1b ubiquitin ligase complex. EMBO J. 30, 3285–3297 (2011). 21772249 10.1038/emboj.2011.243 PMC3160663

[b45] Yin Q. *et al.* E2 interaction and dimerization in the crystal structure of TRAF6. Nat. Struct. Mol. Biol. 16, 658–666 (2009). 19465916 10.1038/nsmb.1605 PMC2834951

[b46] Bellon S. F., Rodgers K. K., Schatz D. G., Coleman J. E. & Steitz T. A. Crystal structure of the RAG1 dimerization domain reveals multiple zinc-binding motifs including a novel zinc binuclear cluster. Nat. Struct. Biol. 4, 586–591 (1997). 9228952 10.1038/nsb0797-586

[b47] Nayak D. & Sivaraman J. Structural basis for the indispensable role of a unique zinc finger motif in LNX2 ubiquitination. Oncotarget 6, 34342–34357 (2015). 26451611 10.18632/oncotarget.5326 PMC4741457

[b48] Vranken W. F. *et al.* The CCPN data model for NMR spectroscopy: development of a software pipeline. Proteins 59, 687–696 (2005). 15815974 10.1002/prot.20449

[b49] Williamson M. P. Using chemical shift perturbation to characterise ligand binding. Prog Nucl Magn Reson Spectrosc 73, 1–16 (2013). 23962882 10.1016/j.pnmrs.2013.02.001

[b50] Isaksson L. *et al.* Highly efficient NMR assignment of intrinsically disordered proteins: application to B- and T cell receptor domains. PLos one 8, e62947 (2013). 23667548 10.1371/journal.pone.0062947 PMC3647075

[b51] Orekhov V. Y. & Jaravine V. A. Analysis of non-uniformly sampled spectra with multi-dimensional decomposition. Prog Nucl Magn Reson Spectrosc 59, 271–292 (2011). 21920222 10.1016/j.pnmrs.2011.02.002

[b52] Kabsch W. XDS. Acta Crystallogr. D Biol. Crystallogr. 66, 125–132 (2010). 20124692 10.1107/S0907444909047337 PMC2815665

[b53] McCoy A. J. *et al.* Phaser crystallographic software. J Appl Crystallogr 40, 658–674 (2007). 19461840 10.1107/S0021889807021206 PMC2483472

[b54] Winn M. D. *et al.* Overview of the CCP4 suite and current developments. Acta Crystallogr. D Biol. Crystallogr. 67, 235–242 (2011). 21460441 10.1107/S0907444910045749 PMC3069738

[b55] Vagin A. A. *et al.* REFMAC5 dictionary: organization of prior chemical knowledge and guidelines for its use. Acta Crystallogr. D Biol. Crystallogr. 60, 2184–2195 (2004). 15572771 10.1107/S0907444904023510

[b56] Chen V. B. *et al.* MolProbity: all-atom structure validation for macromolecular crystallography. Acta Crystallogr. D Biol. Crystallogr. 66, 12–21 (2010). 20057044 10.1107/S0907444909042073 PMC2803126

[b57] Pettersen E. F. *et al.* UCSF Chimera–a visualization system for exploratory research and analysis. J Comput Chem. 25, 1605–1612 (2004). 15264254 10.1002/jcc.20084

