# Supplementary material for: A C2HC zinc finger is essential for the RING-E2 interaction of the ubiquitin ligase RNF125
Source: Sci Rep. 2016 Jul 14;6:29232. doi: 10.1038/srep29232 (PMC4944129; doi:10.1038/srep29232)
Supplement: Supplementary Information [file srep29232-s1.pdf]

SUPPLEMENTARY DATA BELONGING TO MANUSCRIPT:

A C2HC zinc finger is essential for the RING-E2 interaction of the ubiquitin ligase RNF125

Marie-José Bijlmakers[1,] *, João MC Teixeira[2], Roeland Boer[3], Maxim Mayzel[4], Pilar Puig-Sàrries[1], Göran Karlsson[4], Miquel Coll[1,5], Miquel Pons[2], Bernat Crosas[1,] *

1. Institut de Biologia Molecular de Barcelona, CSIC, Parc Cientific de Barcelona, 08028 Barcelona, Spain
2. BioNMR Laboratory, Inorganic and Organic Chemistry Department, Organic Chemistry Section, University of Barcelona, Parc Cientific de Barcelona, 08028 Barcelona, Spain
3. XALOC beamline, ALBA synchrotron (CELLS), 08290 Cerdanyola del Valles, Spain
4. Swedish NMR Centre, University of Gothenburg, SE-40530, Gothenburg, Sweden
5. Institute for Research in Biomedicine (IRB Barcelona), Parc Cientific de Barcelona, 08028 Barcelona, Spain

* Correspondence: mbibmc@ibmb.csic.es/marie.bijlmakers@kcl.ac.uk (MB), bcnbmc@ ibmb.csic.es (BC), Institut de Biologia Molecular de Barcelona, CSIC, Parc Cientific de Barcelona, Calle de Baldiri Reixac 10-12, 08028 Barcelona, Spain. Phone: 00 34 93 4033731

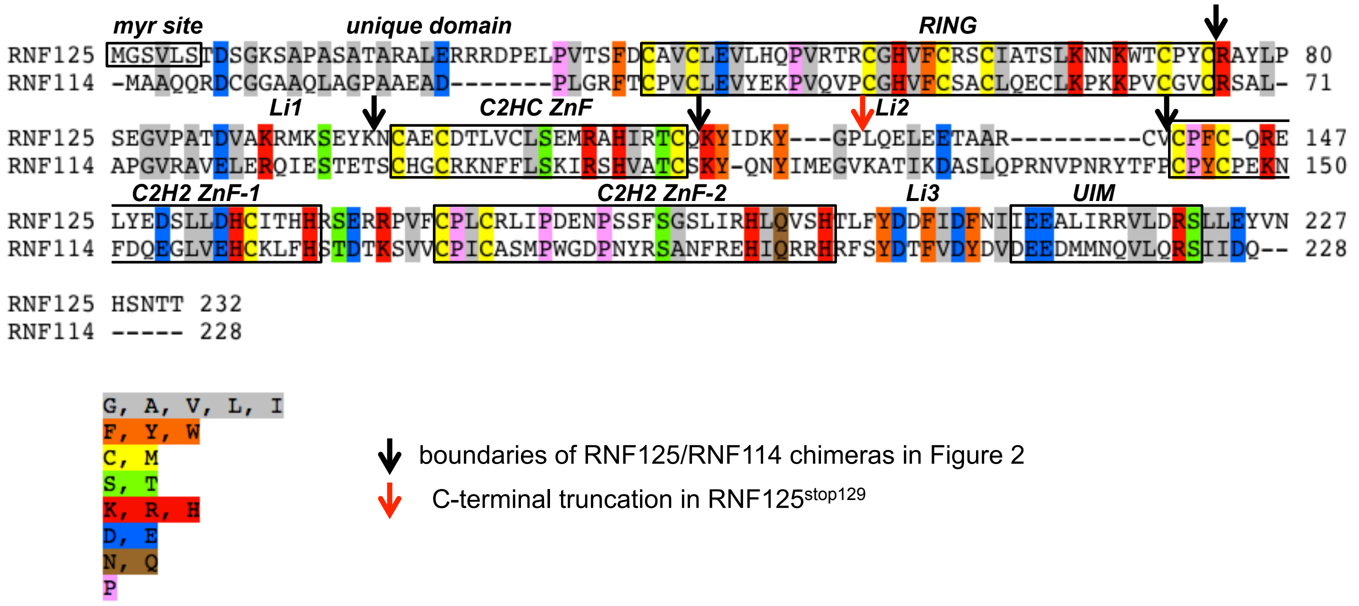

Supplementary Figure S1. Amino acid sequence alignment of human RNF125 and RNF114. The alignment was generated with EMBOSS Needle from EMBL-EBI (www.ebi.ac.uk/Tools/psa/emboss_needle), and coloured using Color Align Properties from the Sequence Manipulation Suite (http://www.bioinformatics.org/sms2/color_align_prop.html, Stothard P (2000) The Sequence Manipulation Suite: JavaScript programs for analyzing and formatting protein and DNA sequences. Biotechniques 28:1102-1104.) This alignment was used to determine RNF125/RNF114 boundaries in the chimeras (indicated by black arrows).

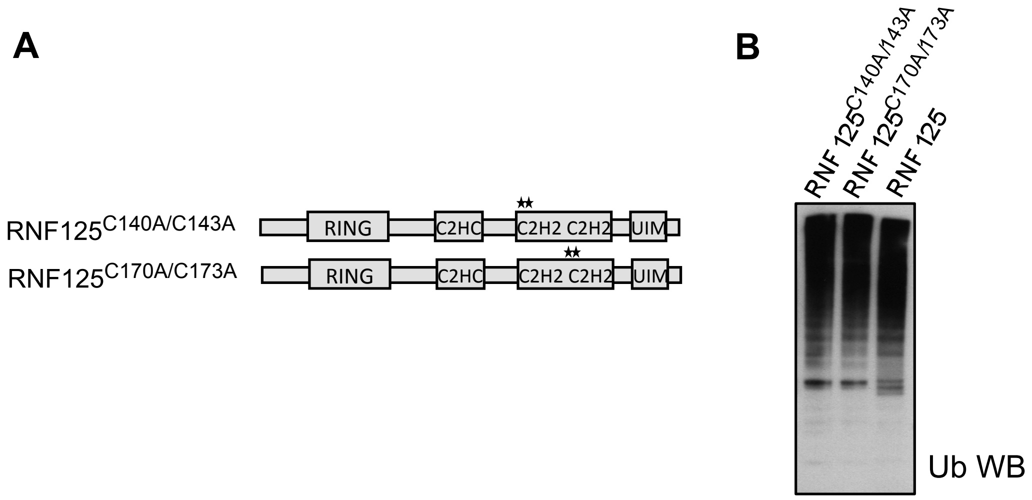

Supplementary Figure S2. Mutations in the second and third ZnFs do not affect activity of RNF125.

A. Diagram of the RNF125 mutants with alanine substitutions at cysteines that are predicted to chelate zinc in the second ($RNF125^{C140A/C143A}$) and third ($RNF125^{C170A/C173A}$) Zn finger (indicated by stars).

B. *In vitro* ubiquitination reactions with RNF125 and the mutants described in A were performed and analyzed as described in Materials and Methods in the main text.

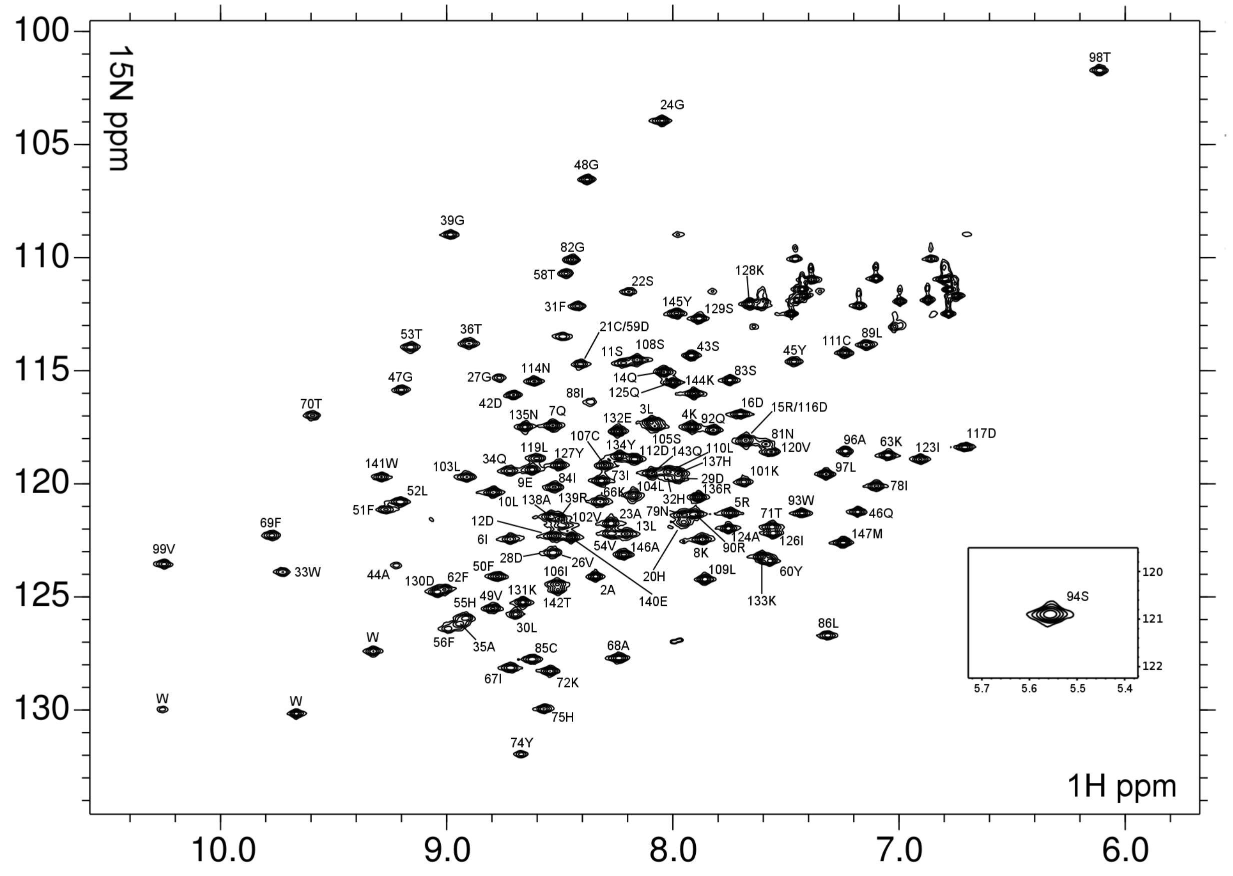

Supplementary Figure S3. Backbone assignment of $^{13}C^{15}N$-UbcH5a.

HSQC spectrum showing the assigned peaks for $^{13}C^{15}N$-UbcH5a. 123 of the 132 peaks for non-proline residues (of 147 amino acids in total) could be assigned. Of these, 102 were assigned automatically. The remainder of the peaks were identified by comparison with BMRB deposited backbone assignments for UbcH5a (Saxena et al., 2005) and the 89% identical protein UbcH5b (Dominguez et al., 2004; Farrow et al., 2000). Since the UbcH5a recording by Saxena et al was performed at pH6.0 instead of pH7.0 in our experiments, we also acquired HSQCs of ^{15}N-UbcH5a at pH6.0, and 6.5 to further aid the assignment. Peaks that were not assigned are M1, A19, I37, M38, S80, D87, S91, S100 and D122. Some of these were not visible at pH 7.0, but could be observed at the lower pHs (S80, D87, S91 and D122). The residues in paired peaks D29 /H32, as well as C21/ D59, were assigned but treated as a combined couple due to uncertainty. The CSPs for these residues were of similar magnitude within each pair and have been included in the analysis represented in Figure 4 since the error associated with mislabelling is negligible.

References:

Saxena, K. et al. (2005). Backbone NMR assignment of the human E2 ubiquitin conjugating enzyme UbcH5alpha (F72K,F82S) double mutant. J Biomol NMR *32(4)*, 338.

Farrow, N. A., et al. (2000). Backbone resonance assignment of human UBC4. J Biomol NMR *18(4)*, 363-364.

Dominguez et al. (2004). Structural model of the UbcH5B/CNOT4 complex revealed by combining NMR, mutagenesis, and docking approaches. Structure *12*, 633-644.

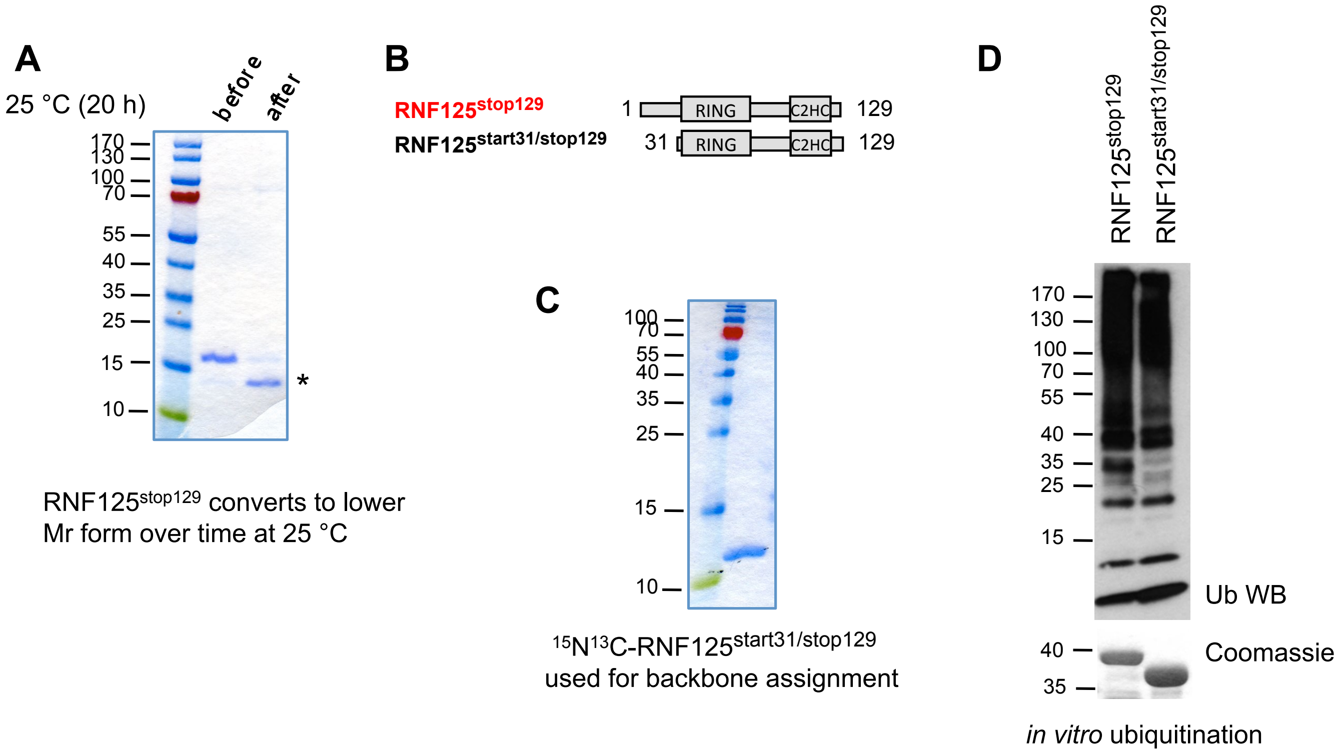

Supplementary Figure S4. properties of RNF125^{stop129} and RNF125$^{start31/stop129}$

A. RNF125^{stop129} (2 μg) was analyzed after purification and storage at -80 °C (before) or after NMR recording and storage at 25 °C for a total of 20 h (after). RNF125^{stop129} converts to a lower Mr protein (*) over time. A Coomassie stained gel is shown.

B. Schematic diagrams of the constructs RNF125^{stop129} and RNF125$^{start31/stop129}$.

C. The purified $^{15}N^{13}C$-RNF125$^{start31/stop129}$ protein that was used for backbone assignment on a Coomassie stained gel.

D. Comparison of the *in vitro* ubiquitination activity of RNF125^{stop129} and RNF125$^{start31/stop129}$. The reactions were performed as described in Materials and Methods, for 60 min at 37 °C, in the presence of UbcH5b. Ubiquitination was detected by Western blotting (WB) for ubiquitin (Ub), input of GST fusion proteins is shown by Coomassie staining.

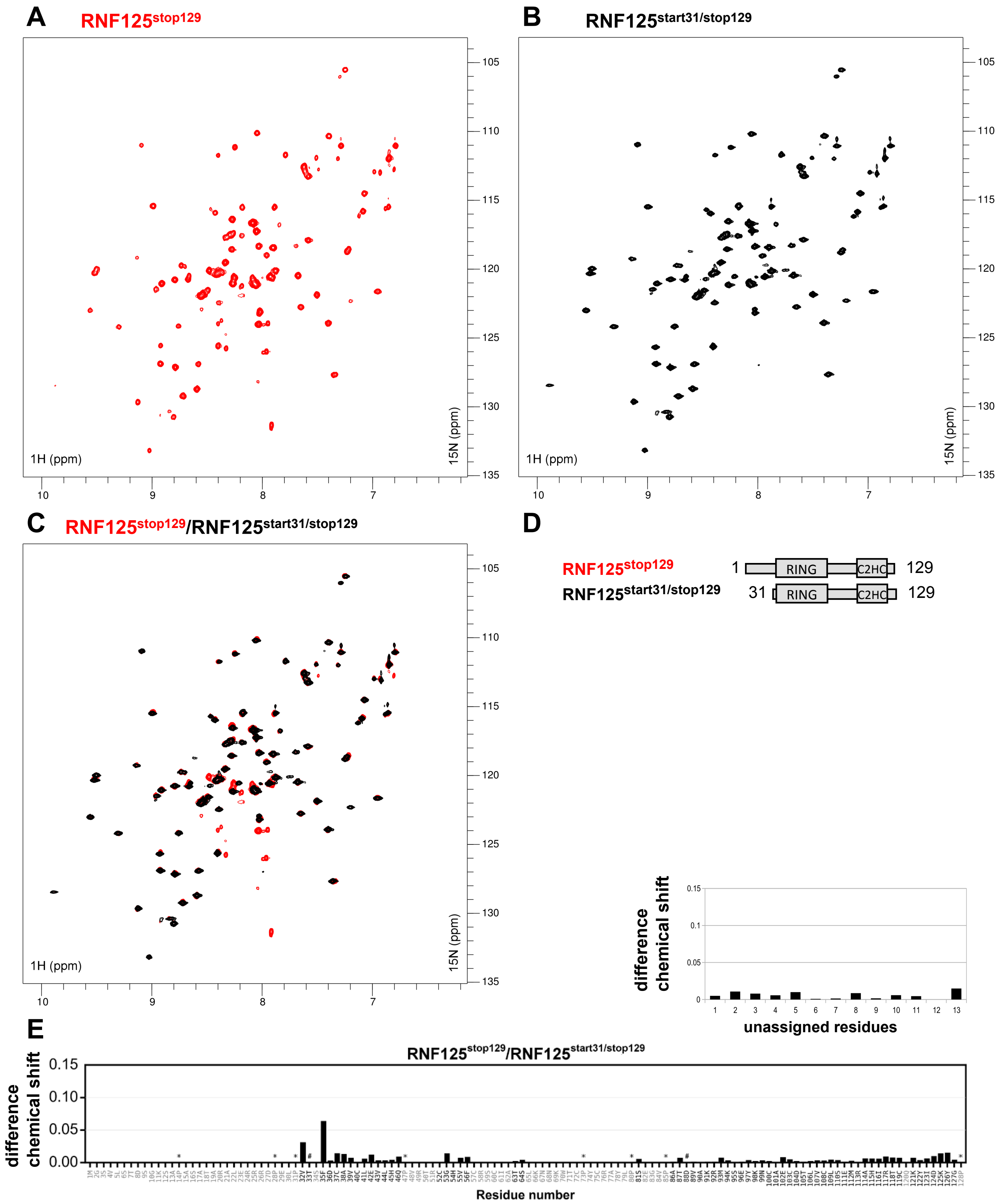

Supplementary Figure S5. HSQCs of ^{15}N-RNF125^{stop129} and ^{15}N-RNF125$^{start31/stop129}$ comparison

A. HSQC of ^{15}N-RNF125^{stop129}

B. HSQC of ^{15}N-RNF125$^{start31/stop129}$

C. An overlay of both HSQCs shows an overlap of peaks, except for those that are absent from the central region of RNF125$^{start31/stop129}$, which represent the N-terminal 30 amino acids that are predicted to be unstructured.

D. Schematic diagrams of the constructs RNF125^{stop129} and RNF125$^{start31/stop129}$.

E. A plot of the differences in chemical shifts between RNF125^{stop129} and RNF125$^{start31/stop129}$. Unassigned residues are those indicated by red boxes in Fig. S7B.

A

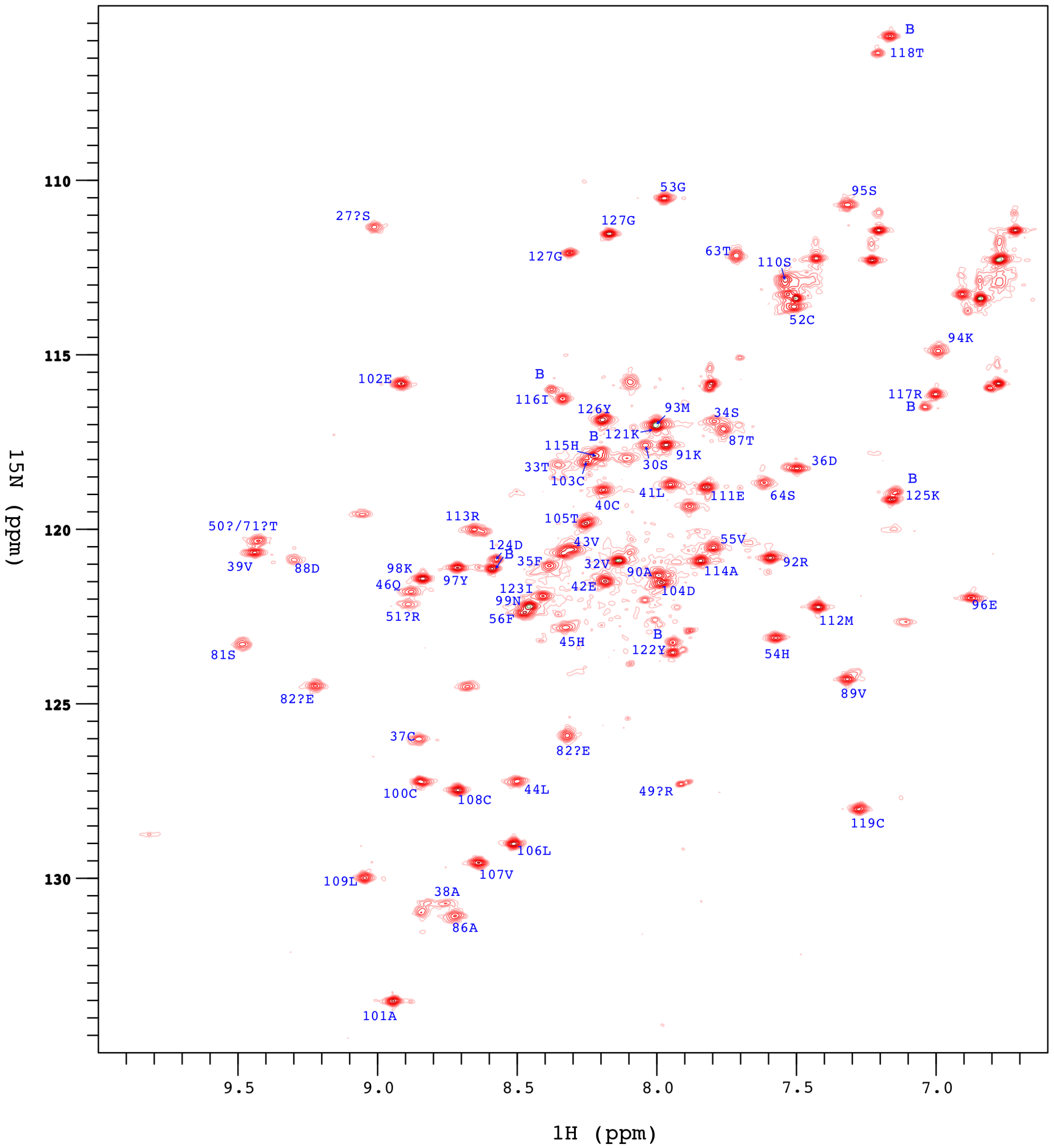

B **Distribution of assigned peaks per domain**

	assigned	unassigned	prolines	total
pGex linker	1	3	1	5
N-terminal region	4	1	1	6
RING	17	20	2	39
Linker 1	15	7	2	24
C2HC ZnF	20	0	0	20
Linker 2 (120-128)	7	1	1	9
	**64**	**32**	**7**	**103**

Supplementary Figure S6. Backbone assignment of RNF125$^{start31/stop129}$

A. ^{1}H-^{15}N HSQC spectrum of RNF125$^{start31/stop129}$ assignment sample. C103, I116, R117, T118, Y122, D124, K125 and G127 appear as double peaks, the minor species is labelled "B". 27S and 30S are residues of the pGex linker.

B. A table showing the distribution of assigned peaks per domain.

C

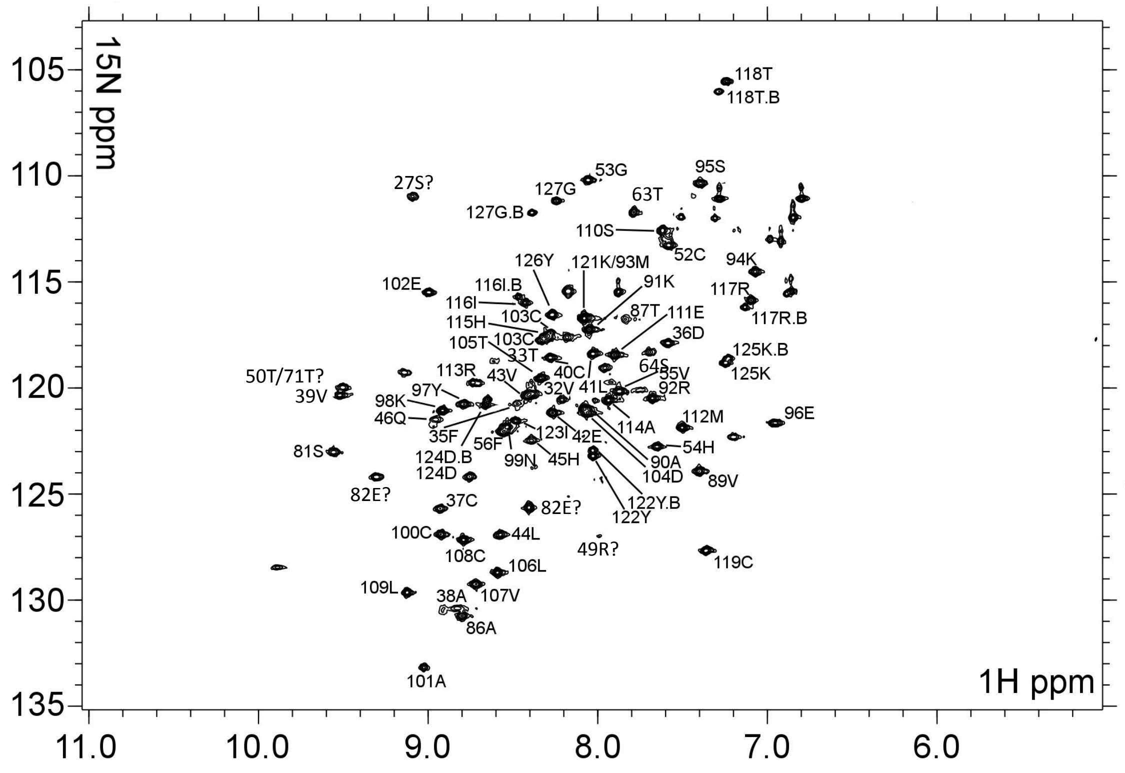

D

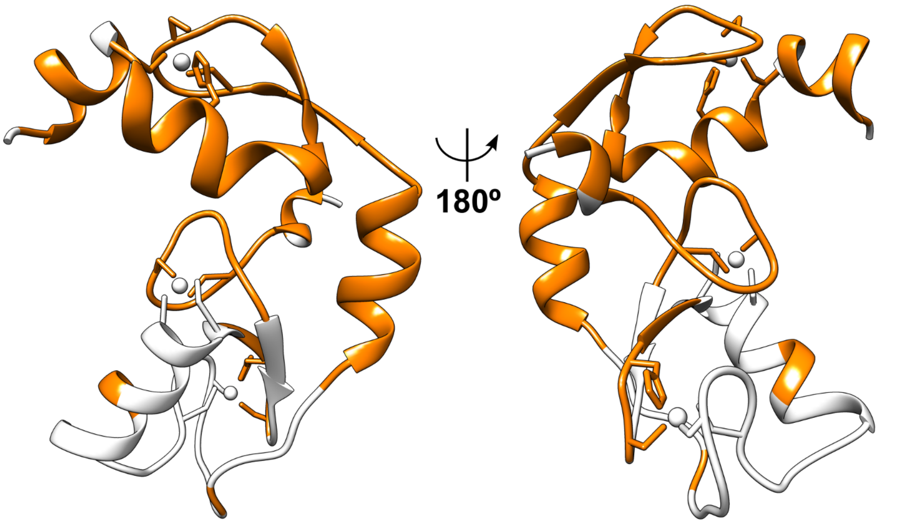

E

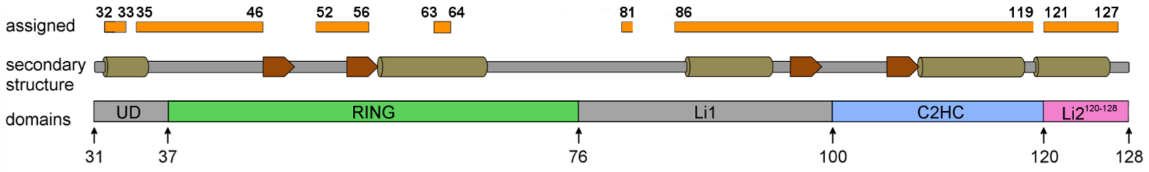

Supplementary Figure S6 continued. Backbone assignment of RNF125$^{start31/stop129}$

C. Assigned peaks for reference spectrum of ^{15}N-RNF125$^{start31/stop129}$ used in UbcH5a titration experiments in Fig. 5. Buffer conditions were slightly different from assignment sample (50 mM Tris PH 7.4, 150 mM NaCl, 200 μM $ZnCl_2$ versus 50 mM Tris PH 7.0, 50 mM NaCl, 200 μM $ZnCl_2$ for assignment), which did not affect the majority of the peaks but small differences can be observed.

D. Crystal structure of RNF125$^{start31/stop129}$ with assigned residues in orange.

E. Schematic diagrams of RNF125$^{start31/stop129}$ with assigned regions in orange.

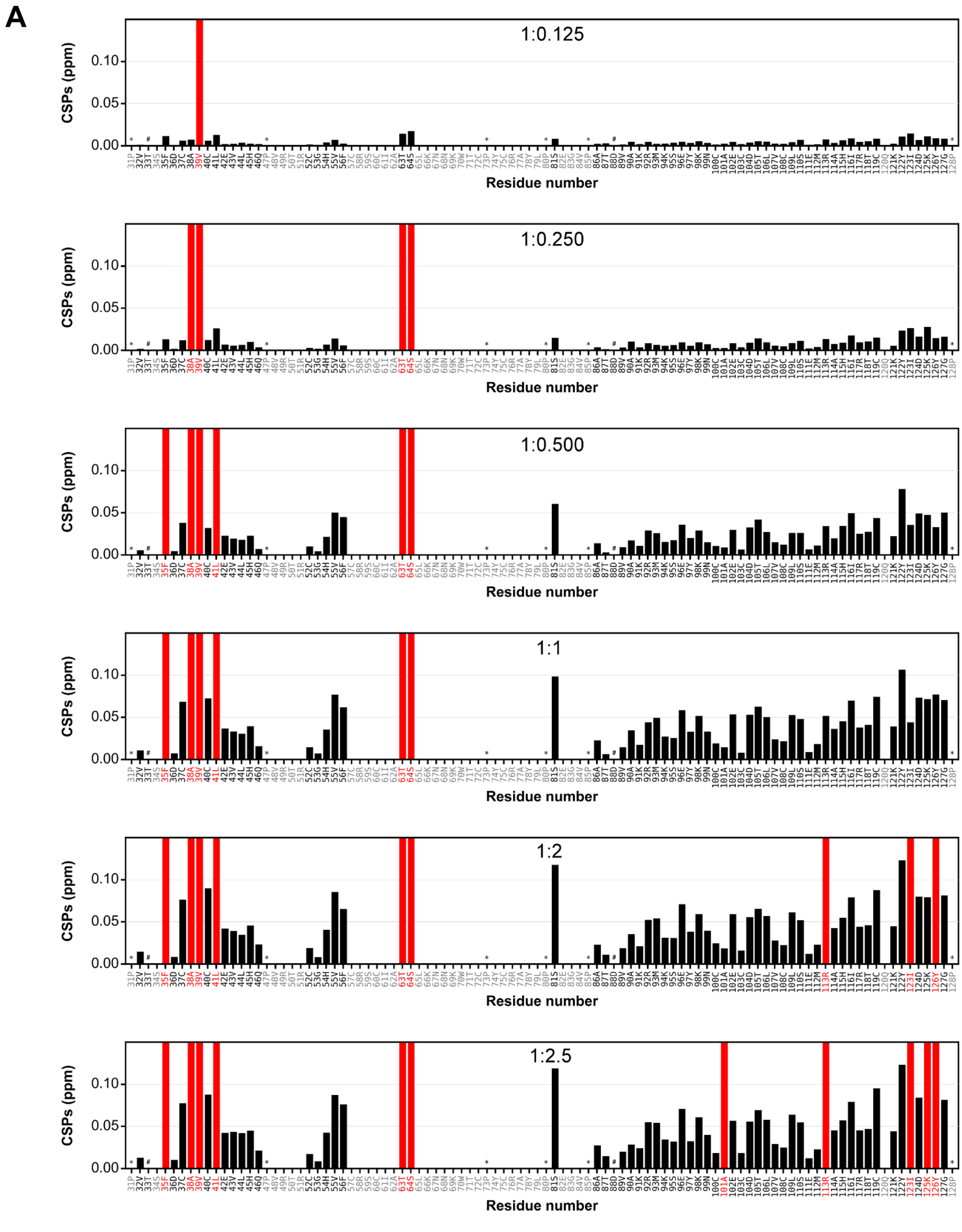

Supplementary Figure S7. CSPs of ^{15}N-RNF125$^{start31/stop129}$ caused by titration of UbcH5a.
A. HSQC spectra of 0.2 mM ^{15}N-RNF125$^{start31/stop129}$ were recorded before and after the addition of UbcH5a at molar equivalents ranging from 0.125 to 2.5 as indicated. Combined ^{1}H and ^{15}N CSPs for each titration point are plotted. Details are as in Figure 5 of the main text where the data for 1:2 (^{15}N-RNF125$^{start31/stop129}$/UbcH5a) of this experiment are represented. Red bars represent residues that broadened beyond detection in the presence of UbcH5a.

B

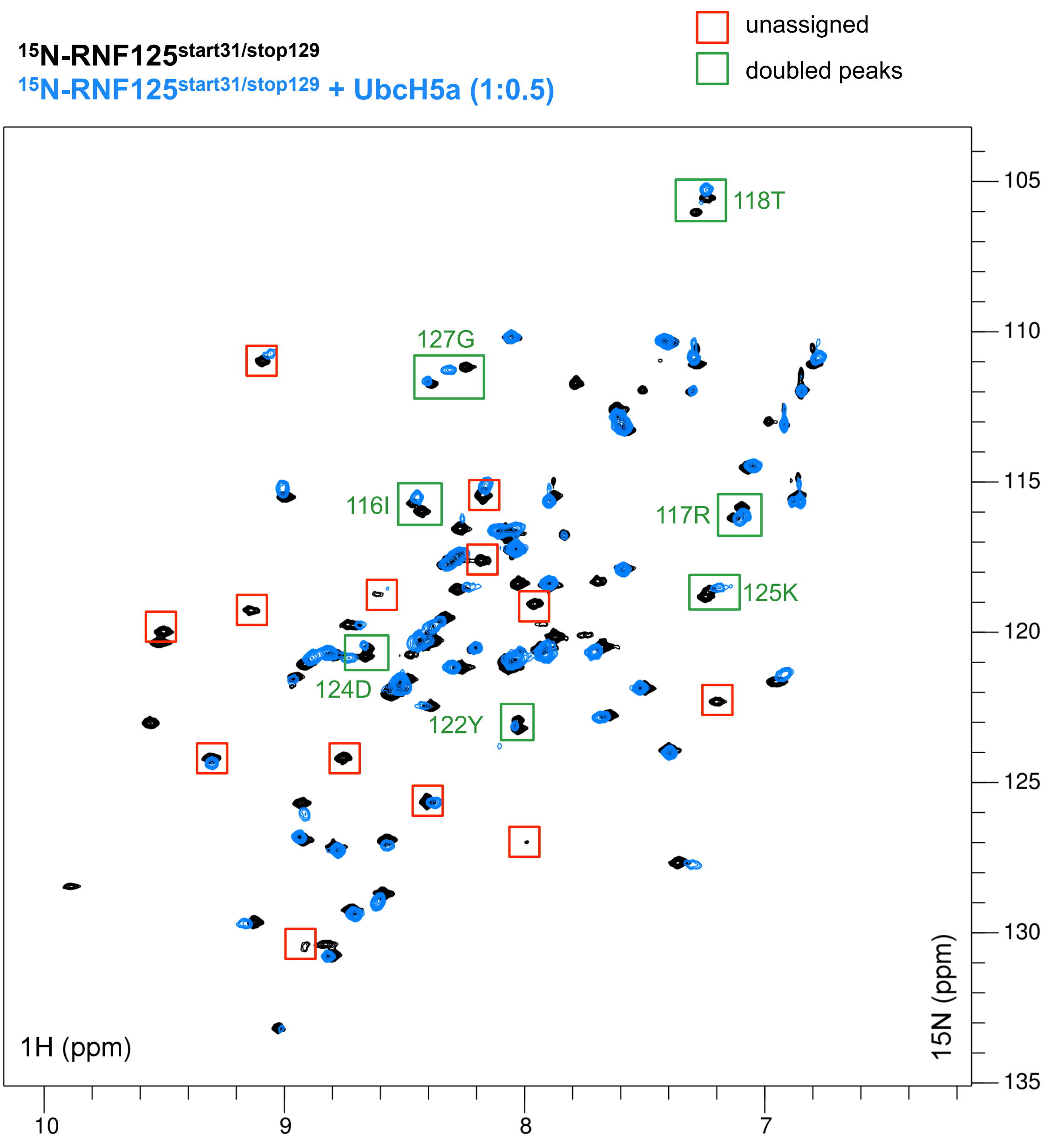

Supplementary Figure S7 continued. CSPs in ^{15}N-RNF125$^{start31/stop129}$ caused by titration of UbcH5a. Highlighting of unassigned sharp peaks and doubled peaks.

B. HSQC spectrum of 0.2 mM ^{15}N-RNF125$^{start31/stop129}$ before (black) and after (blue) the addition of 0.5 molar equivalents of UbcH5a. Sharp unassigned peaks (in red boxes) undergo chemical shifts or peak broadening as well. Residues with doubled peaks are labelled as well (green boxes). For these, CSPs at both major and minor peaks are detected in the presence of UbcH5a.

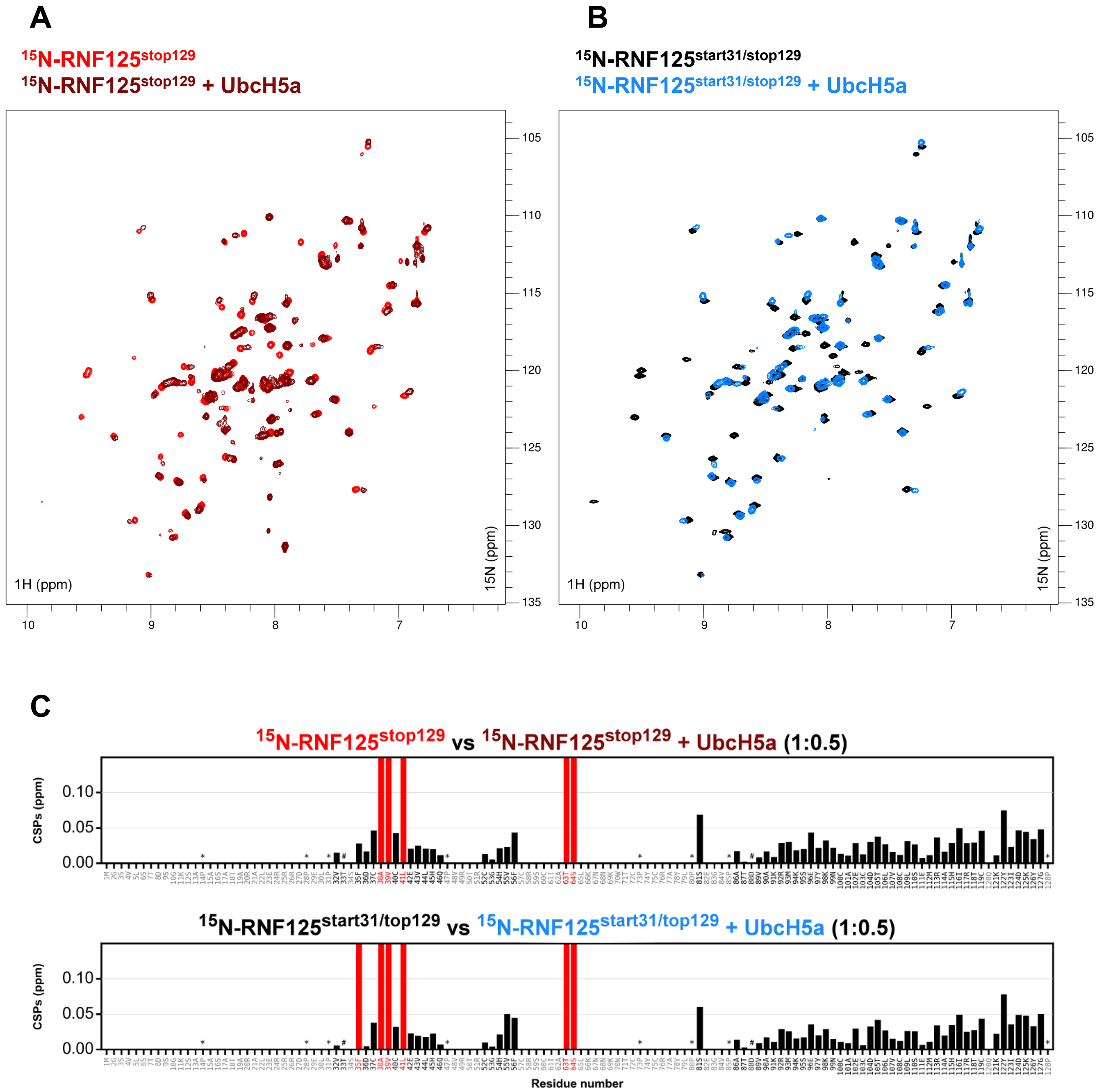

Supplementary Figure S8. Comparison of CSPs for ^{15}N-RNF125^{stop129} and ^{15}N-RNF125$^{start31/stop129}$ in the presence of UbchH5a

A. Overlay of ^{1}H-^{15}N HSQC spectra of 0.2 mM ^{15}N-RNF125^{stop129} collected at 600 MHz before (red), and after addition of 0.5 molar equivalents of UbcH5a (maroon).

B. Overlay of ^{1}H-^{15}N HSQC spectra of 0.2 mM ^{15}N-RNF125$^{start31/stop129}$ collected at 600 MHz before (black), and after addition of 0.5 molar equivalents of UbcH5a (blue).

C. Plot of the combined 1H and 15N chemical shift perturbations for ^{15}N-RNF125^{stop129} (top) and ^{15}N-RNF125$^{start31/stop129}$ (bottom) at 0.5 molar equivalents of UbcH5a. Assigned residues are in black in the sequence underneath the graph, prolines are indicated by (*). Red bars represent residues that broadened beyond detection in the presence of UbcH5a.

A

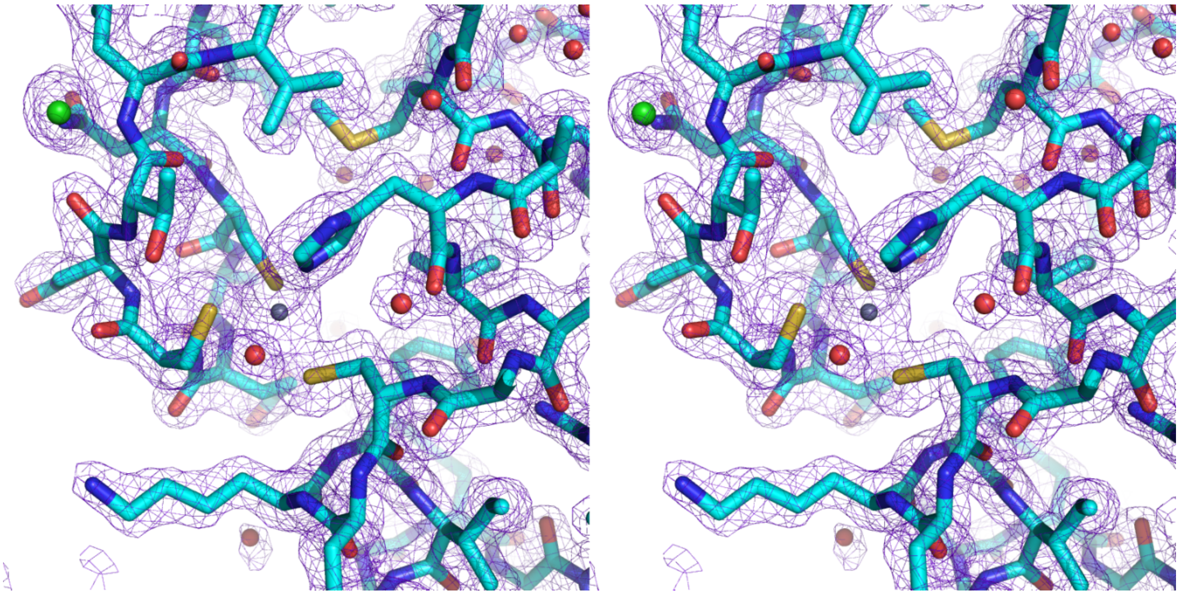

B

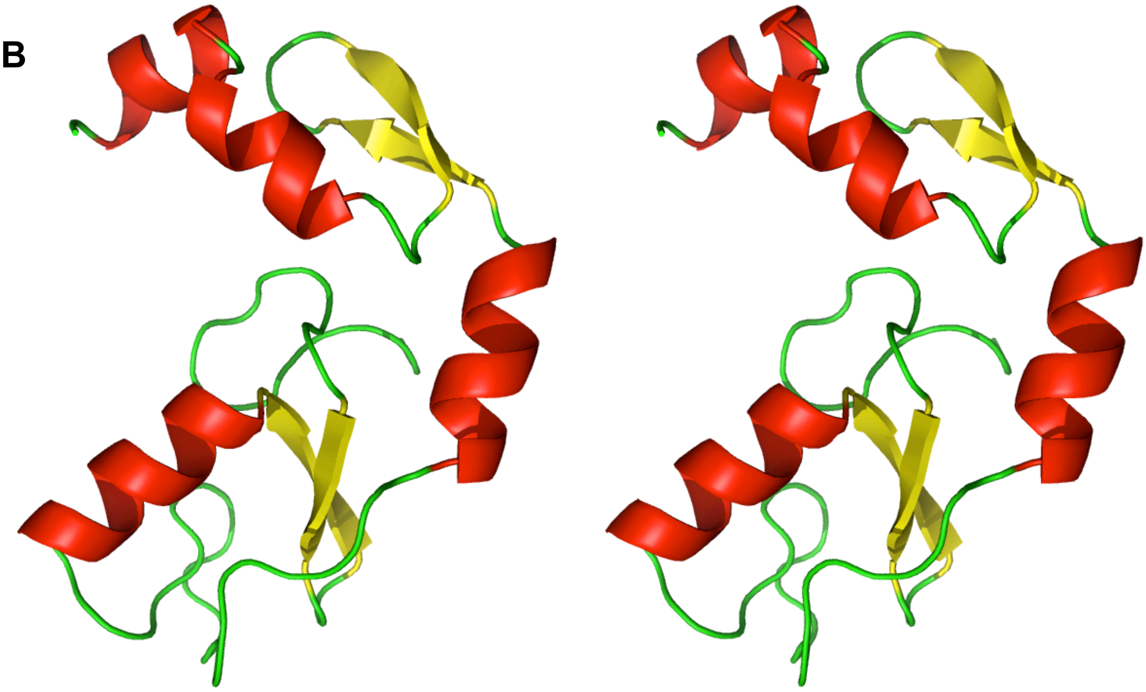

Supplementary Figure S9. Crystal structure of RNF125$^{start31/stop129}$

A. A stereo view on the coordinated Zn atom of the Zn finger, showing the weighted $2F_o$-F_c electron density map at 1.0σ. Water molecules, Zn atoms and Cl^- ions are represented by red, grey and green spheres, respectively.

B. stereo view of the RNF125^{stop129} structure as a ribbon diagram.

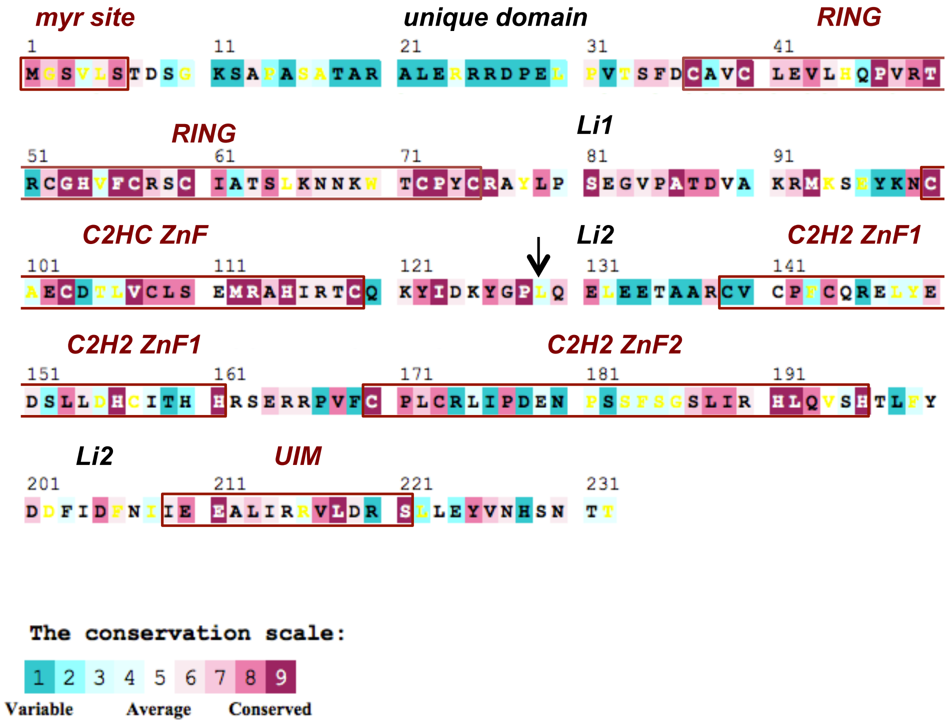

Supplementary Figure S10. Conservation score for RNF125 amino acids

42 unique RNF125 sequences were used for conservation score calculation by ConSurf (www.consurf.ta.ac.il. Celniker et al, Isr. J. Chem. 2013 March 10, doi: 10.1002/ijch.201200096, Ashkenazy et al, 2010, Nucl. Acids Res. 2010; DOI: 10.1093/nar/gkq399).

Residues for which the conservation score was below the confidence cut-off are in yellow.

The black arrow indicats the last residue, P128, of the truncated RNF125^{stop129}.

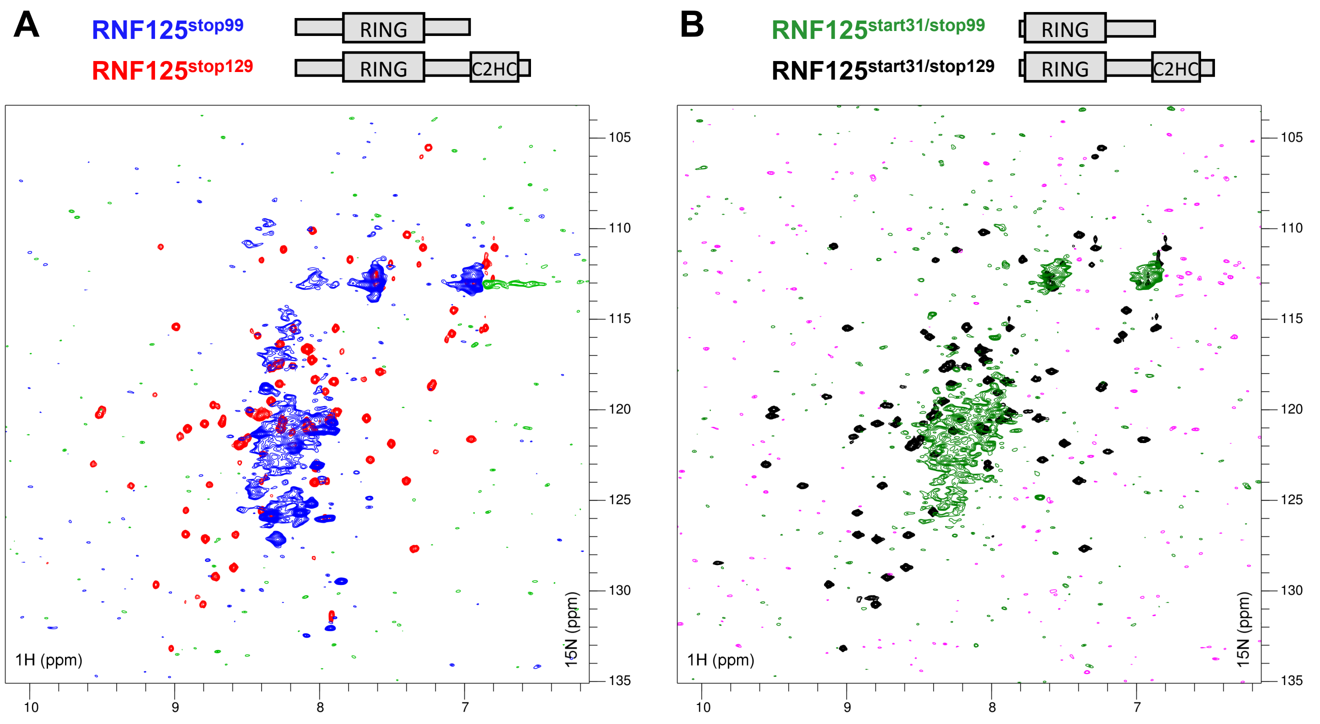

Supplementary Figure S11. The RNF125 RING domain lacks structure in the absence of the C2HC ZnF.

A. Overlay of HSQCs for the ^{15}N-RNF125^{stop99} construct (blue) without, and the ^{15}N-RNF125^{stop129} with the C2HC ZnF (red). The lack of well-dispersed peaks from the RNF125^{stop99} HSQC indicates that the RING domain is mostly unfolded in the absence of the C2HC ZnF.

B. Overlay of HSQCs for ^{15}N-RNF125$^{start31/stop99}$ (green) and ^{15}N-RNF125$^{start31/stop129}$ (black). These constructs are similar to those in A, but start at amino acid 31 (see diagrams). As in A) for RNF125^{stop99}, very few well-dispersed peaks are observed for RNF125$^{start31/stop99}$, again indicative of a lack of structure for the RING domain.

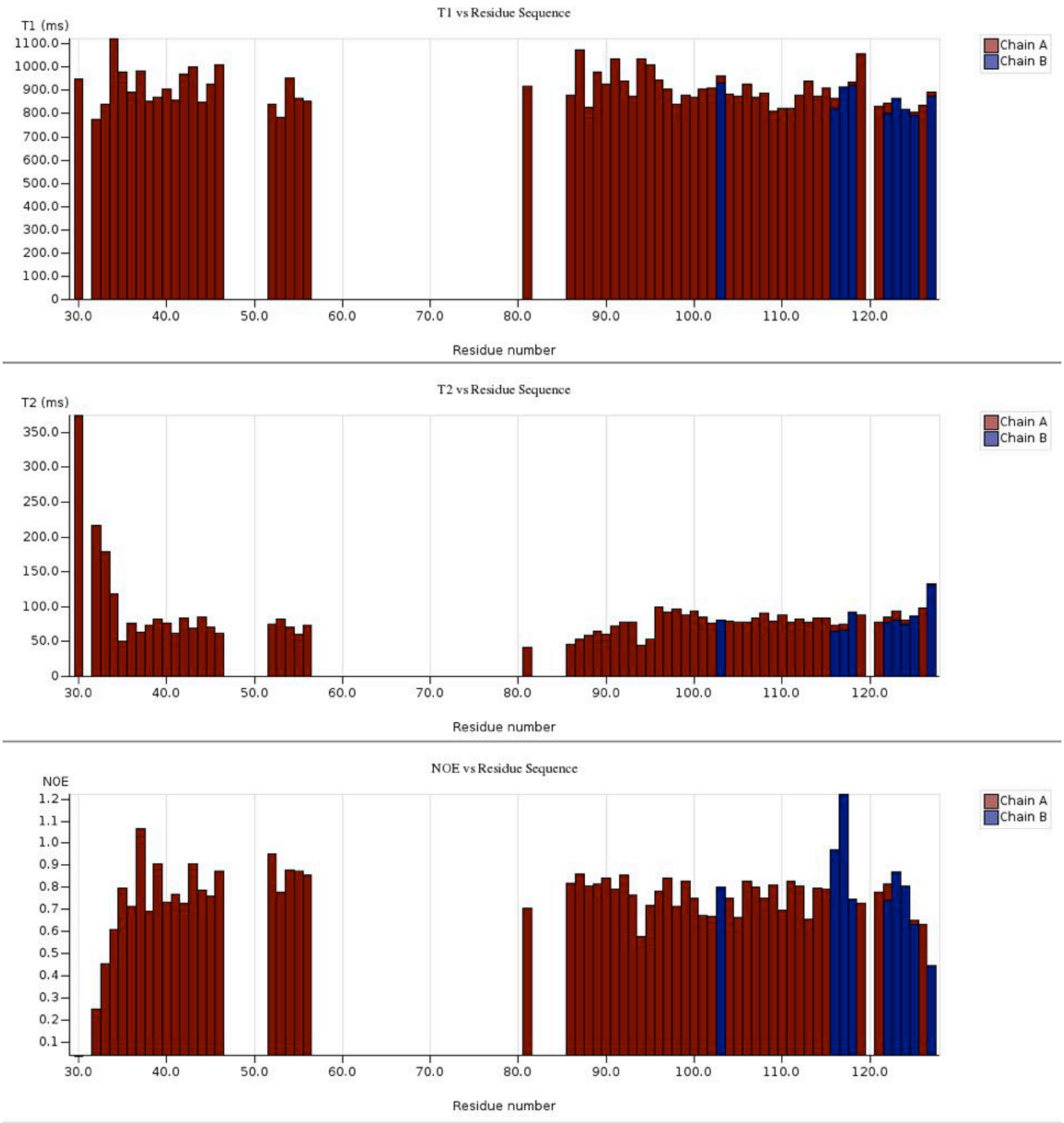

Supplementary Figure S12. NMR relaxation experiments of RNF125$^{start31/stop129}$

T1, T2 and NOE values acquired by NMR spectroscopy for ^{15}N-RNF125$^{start31/stop129}$ are plotted for individual assigned residues using CCPN Analysis. Chain B refers to the minor species for the residues that are found in two different conformations (see Figures 5 and S6). The estimated rotational correlation time calculated from the T1/T2 ratio was ~7.5 ns, which corresponds to the correlation time of a protein with a Mw of 11-12 kDa. Since the calculated Mw of ^{15}N-RNF125$^{start31/stop129}$ is 11.5 kD, it can be concluded that this protein is present as a monomer in solution.
